# Modulation of gut microbiota and delayed immunosenescence as a result of syringaresinol consumption in middle-aged mice

**DOI:** 10.1038/srep39026

**Published:** 2016-12-15

**Authors:** Si-Young Cho, Juewon Kim, Ji Hae Lee, Ji Hyun Sim, Dong-Hyun Cho, Il-Hong Bae, Hyunbok Lee, Min A. Seol, Hyun Mu Shin, Tae-Joo Kim, Dae-Yong Kim, Su-Hyung Lee, Song Seok Shin, Sin-Hyeog lm, Hang-Rae Kim

**Affiliations:** 1 R&D Unit, AmorePacific Corporation, Gyeonggi-do 17074, Republic of Korea; 2 Department of Anatomy and Cell Biology, Seoul National University College of Medicine, Seoul 03080, Republic of Korea; 3 Department of Biomedical Sciences, and Seoul National University College of Medicine, Seoul 03080, Republic of Korea; 4 BK21Plus Biomedical Science Project, Seoul National University College of Medicine, Seoul 03080, Republic of Korea; 5 College of Veterinary Medicine, Seoul National University, Seoul 08826, Republic of Korea; 6 Academy of Immunology and Microbiology (AIM), Institute for Basic Science (IBS), Pohang, 37673, Republic of Korea; 7 Division of Integrative Biosciences and Biotechnology (IBB), Pohang University of Science and Technology, Pohang, 37673, Republic of Korea

## Abstract

Age-associated immunological dysfunction (immunosenescence) is closely linked to perturbation of the gut microbiota. Here, we investigated whether syringaresinol (SYR), a polyphenolic lignan, modulates immune aging and the gut microbiota associated with this effect in middle-aged mice. Compared with age-matched control mice, SYR treatment delayed immunosenescence by enhancing the numbers of total CD3^+^ T cells and naïve T cells. SYR treatment induced the expression of Bim as well as activation of FOXO3 in Foxp3^+^ regulatory T cells (Tregs). Furthermore, SYR treatment significantly enhanced the *Firmicutes*/*Bacteroidetes* ratio compared with that in age-matched controls by increasing beneficial bacteria, *Lactobacillus* and *Bifidobacterium*, while reducing the opportunistic pathogenic genus, *Akkermansia*. In addition, SYR treatment reduced the serum level of lipopolysaccharide-binding protein, an inflammatory marker, and enhanced humoral immunity against influenza vaccination to the level of young control mice. Taken together, these findings suggest that SYR may rejuvenate the immune system through modulation of gut integrity and microbiota diversity as well as composition in middle-aged mice, which may delay the immunosenescence associated with aging.

Aging is a complex process that involves progressive functional decline in a variety of organ systems, influenced by environmental, stochastic, and (epi)genetic events, and their interactions throughout life^1^. A common feature of aging in tissues and aging-related diseases is chronic inflammation, termed “inflammaging,” describing the low-grade, persistent, systemic inflammation in aging, in the absence of overt infection leading to tissue degeneration and chronic disease^2^. In addition, aging of the immune system is characterized by a progressive decline in adaptive immune function, contributing to increased susceptibility to infections, cancer, autoimmunity, and poor responses to vaccination in the elderly^3^,^4^,^5^,^6^. Thus, interventions designed to reduce chronic inflammation while maintaining an effective adaptive response to insults, such as tissue injury and pathogen exposure, will have wide-ranging benefits for health and lifespan. The hallmarks of immune dysfunction with age include: (i) thymic involution; (ii) reduced T-cell function, including decreased numbers and percentages of peripheral naïve T cells, and accumulation of memory T cells^7^; (iii) increased regulatory T cells (Treg)^8^; (iv) altered frequencies of B cells and reduced antibody production^9^,^10^; and (vi) dysregulated production of soluble mediators, such as cytokines and chemokines^11^. Several interventions have been shown to exert some beneficial effects on the immune system during aging, including caloric restriction (CR)^12^, certain nutrients^13^,^14^, and probiotics^15^. Among them, caloric restriction partially retards or restores age-associated immunosenescence by preserving naïve T cells into old age in nonhuman primates^16^, altering thymus involution^17^, and reducing the production of proinflammatory cytokines^18^. Although the actual mechanism by which CR exerts beneficial effects on the immune system during aging is unknown, the underlying mechanisms of CR are multifaceted, involving energy metabolism, oxidative stress regulation, and neuroendocrine homeostasis^19^.

Recent studies suggested that maintenance of a “youthful” or “healthy” gut microbiota architecture during aging may delay or limit immunosenescence^20^. The profile of the gut microbiota changes with aging, and these changes have been linked to declines in immunity, as observed in immunosenescence. In fact, probiotics and prebiotics downregulate the proinflammatory response^21^,^22^ and improve innate immune dysfunction in the elderly^23^. CR also modulates the structure of the gut microbiota to a more balanced state, thereby alleviating metabolic syndrome during aging^24^. However, the involvement of gut microbiota composition in the progression of age-related immunosenescence remains unclear.

Syringaresinol (SYR; phenol, 4,4′-(tetrahydro-1H,3H-furo[3,4-c]furan-1,4-diyl)bis[2,6-dimethoxy-(1R,3aS,4R,6aS)-rel-]) is a lignan occurring in plant foods, such as oilseeds, cereal brans, and various berry seeds^25^. SYR has been reported to exert various health-promoting effects, including antioxidant, antistress, antitumorigenic, and anti-inflammatory effects^26^,^27^,^28^. These diverse bioactivities of SYR, like other dietary polyphenols, depend on bacterial transformation by the gut microbiota^29^. In turn, SYR may influence the relative abundance of different bacterial groups within the gut microbiota. However, to our knowledge, there have been no reports regarding SYR metabolism by gut microbiota and the effects of SYR on modulation of the gut microbiota. Recently, we demonstrated that SYR delays cellular senescence and alleviates hypoxia/reoxygenation injury through the activation of FOXO3^30^,^31^. SYR stimulated the nuclear translocation of FOXO3, leading to the transcriptional activation of its target genes involved in the antioxidant and anti-inflammatory effects^30^. FOXO can induce tolerance to oxidative stress-induced damage and extend lifespan in vertebrates^32^. FOXO also has diverse functions in immune regulation, such as effects on the frequency of naïve T cells^33^, helper T-cell quiescence^34^, and neutrophil survival^35^. Based on these reports and our previous results, we hypothesized that SYR would delay immunosenescence during aging. Thus, we investigated the impact of SYR feeding on the immune system and gut microbiota architecture during aging. Our data suggest that SYR may delay immunosenescence through modulation of the immune system and the composition and diversity of gut microbiota.

## Results

### SYR treatment restores age-related dysregulation of lymphocyte subsets and effector function

**Figure 1:**
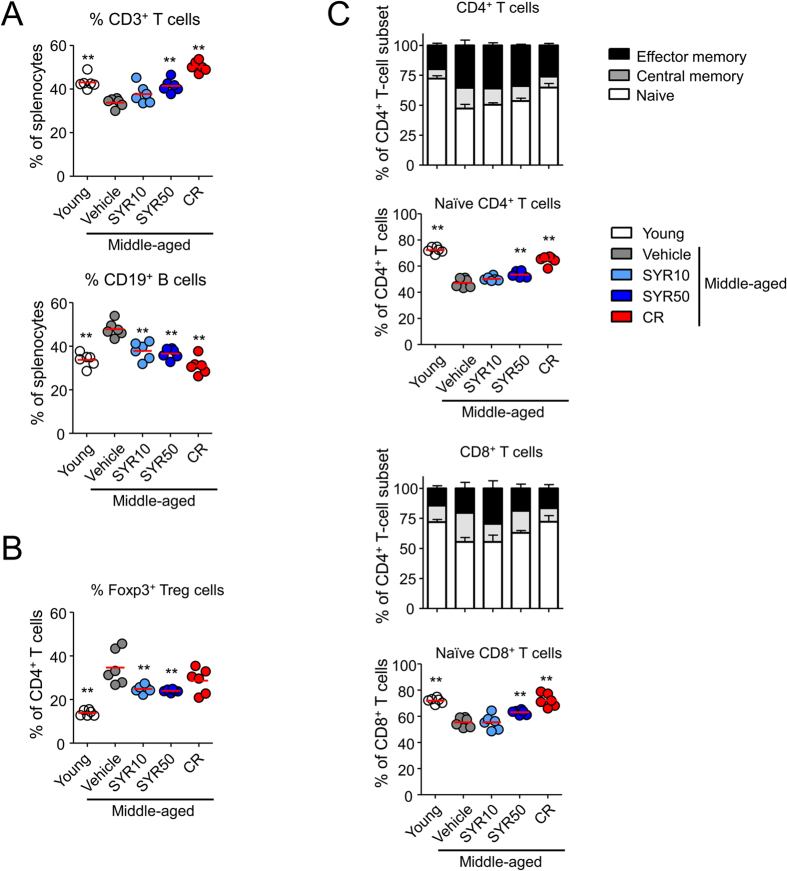
Effects of SYR and CR on the frequencies of splenic lymphocyte subpopulations with aging. Middle-aged mice (44 weeks old) were treated with vehicle (control), 10 mg/kg SYR (SYR10), 50 mg/kg SYR (SYR50), or CR for 10 weeks and their splenocytes were analyzed by flow cytometry. Young mice (10 weeks old) were used as age-mismatched controls. (**A**) Frequencies of CD3^+^ T and CD19^+^ B cells in splenocytes. (**B**) Frequency of Foxp3^+^ Treg in CD4^+^ T cells. (**C**) Distributions of CD4^+^ and CD8^+^ T cell subsets, such as naïve, central memory (CM), and effector memory (EM) T cells. All data are presented as means ± SEM of six mice per group; *P < 0.05, **P < 0.01, ***P < 0.001 versus the untreated middle-aged control group (ANOVA test). Experiments were repeated three times. Data are the averages of three independent experiments.

**Figure 2:**
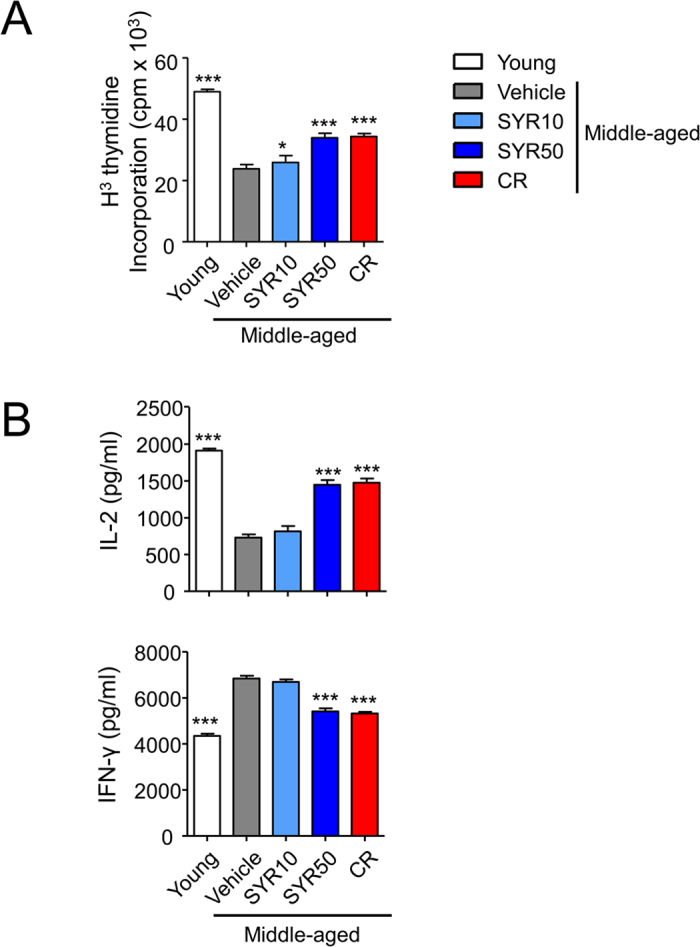
Improvement of the age-related dysfunction of splenic T cells by SYR50 and CR. Middle-aged mice (44 weeks old) were treated with vehicle (control), 10 mg/kg SYR (SYR10), 50 mg/kg SYR (SYR50), or CR for 10 weeks. Young mice were used as age-mismatched controls. (**A**) Splenic T cells from young, middle-aged mice subjected to vehicle, SYR10 or SYR50, and CR were stimulated with anti-CD3 and anti-CD28 Abs for 3 days. Prior to harvesting, cells were incubated overnight in the presence of ^3^H-thymidine, and incorporation of radioactivity was measured. (**B**) Cytokine levels in culture supernatants of splenic T cells were measured using the Luminex System. Results are means ± SEM of three independent experiments performed in quadruplet, each experiment using cells pooled from at least three mice per group; *P < 0.05, **P < 0.01, ***P < 0.001 versus the untreated middle-aged control group.

In middle-aged mice, considerable changes in T-cell subsets are closely related to a shorter lifespan^36^,^37^, and analysis of T-cell subsets provides an indirect measure of the aging rate. To examine the effects of SYR treatment on the immune system in middle-aged mice, 10-month-old mice were orally administered a high (50 mg/kg) (SYR50) or low (10 mg/kg) (SYR10) dose of SYR once daily for 10 weeks. As controls, mice were fed PBS or subjected to a calorie-restricted diet (30% fewer calories, CR), which is known to delay immunosenescence^16^. Consistent with previous results^38^, CR mice exhibited significant declines in blood glucose level as well as body weight (Table S1). Although not statistically significant, mice treated with a high dose of SYR (SYR50) exhibited a slightly reduced blood glucose level without changes in body weight compared with age-matched controls (vehicle) (Table S1). We next compared the absolute numbers and frequencies of splenic leukocyte subpopulations among the treated groups, namely, young (10 weeks old), CR, low- or high-dose SYR-treated middle-aged mice and age-matched mock control mice. In agreement with previous reports^8^,^10^,^39^,^40^, middle-aged mice (vehicle) showed decreases in the frequency of CD3^+^ T cells, and increases in the frequencies of Foxp3^+^ Tregs and CD19^+^ B cells compared with young mice (Fig. 1A and B, Tables S2 and S3). Compared with age-matched controls, mice treated with SYR50 showed enhanced numbers of total CD3^+^ T cells and naïve T cells in both CD4^+^ and CD8^+^ populations (Tables S2 and S3 and Fig. 1C). CR also alleviated the age-related decline in T-cell frequency, especially naïve CD4^+^ and naïve CD8^+^ cells (Fig. 1A–C). Interestingly, SYR treatment lowered the frequency of CD4^+^ Foxp3^+^ Treg cells (Fig. 1B) without altering the numbers of dendritic cells or monocytes (Tables S2 and S3). However, SYR treatment did not reverse the frequencies of B-cell subsets, marginal zone B, follicular B, or transitional B cells (Tables S2 and S3). We also analyzed the effector function and proliferative activity of the T cells among the treatment groups as the results of simple analyses of lymphocyte populations are not always correlated with “younger” phenotypes in immune function in the elderly^41^,^42^. To examine the proliferative response, we performed a ^3^H thymidine incorporation assay on splenic T cells stimulated with anti-CD3/anti-CD28 antibodies (Abs). The proliferative capacity of T cells from the middle-aged control mice was significantly lower (*p* < 0.0001) than that of young mice (Fig. 2A), while impaired proliferation upon TCR stimulation with anti-CD3/CD28 Abs was markedly improved by administration of SYR50 as well as by CR. As age-dependent dysregulation of cellular immunity is closely related to alteration of cytokine expression^43^,^44^, we measured the levels of diverse cytokines. Compared with young mice, middle-aged mice produced lower levels of IL-2 but showed enhanced IFN-γ production (Fig. 2A). SYR50 treatment and CR prevented these age-associated alterations in IL-2 and IFN-γ production (Fig. 2B). Taken together, these findings suggest that SYR may delay immunosenescence by modulating the frequencies/numbers of naïve and regulatory T lymphocytes and by enhancing cellular immunity in middle-aged mice, comparable to the levels of middle-aged mice subjected to CR.

### SYR induces Bim expression and FOXO3 activation in Tregs from middle-aged mice

**Figure 3:**
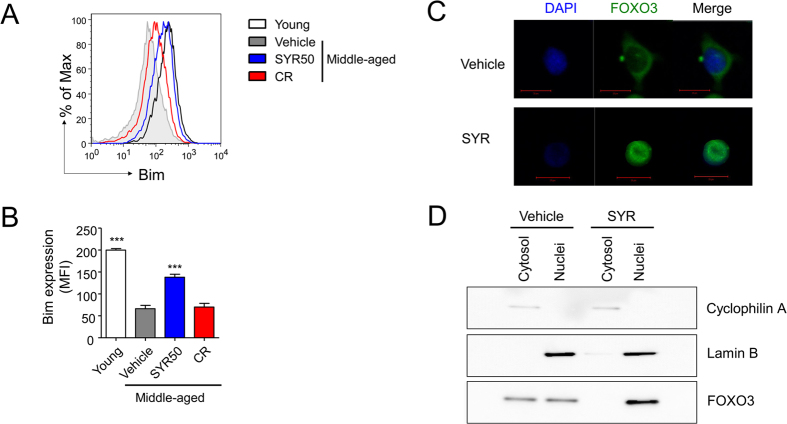
Reversal of Bim expression and redistribution of FOXO3 in Tregs by SYR50 treatment. Splenocytes from young mice (10 weeks old) or middle-aged mice subjected to vehicle (control), 50 mg/kg SYR, or CR (each group *n* = 3) were stained with Abs against CD4, intracellular foxp3, and Bim, and analyzed by flow cytometry. (**A**) Representative histogram and (**B**) bar graph showing Bim expression in Treg cells from each group. (**C**) Cellular localization of FOXO3 was determined by immunofluorescence. Sorted CD4^+^CD25^+^ Tregs were fixed and stained with anti-FOXO3 (green) and DNA was stained with 4′,6-diamidino-2-phenylindole (DAPI) (blue). Bar: 20 μm. (**D**) Equal cell equivalents of nucleus and cytosol were separated by gel electrophoresis and processed for Immunoblotting with the indicated antibodies. All results are either representatives or means ± SEM of three independent experiments; ***P < 0.001 versus the untreated middle-aged control group.

Significant increases in Treg cell number with aging markedly contribute to impaired T-cell responses in aged hosts in both mice and humans^45^. Accumulation of Foxp3^+^ Treg cells with aging is the result of enhanced survival by decreased expression of Bim, a proapoptotic BH3-only Bcl-2 family member^46^. We examined whether decreased frequency of CD4^+^ Foxp3^+^ Treg cells by SYR treatment (Fig. 1B) is related to alteration of Bim expression in Treg cells. Consistent with previous reports^46^, Bim expression was decreased by about threefold in Tregs of middle-aged mice compared with that in young mice (Fig. 3A and B). Interestingly, SYR50 treatment significantly enhanced Bim expression in Tregs to levels similar to those seen in young mice, while CR did not affect Bim expression (Fig. 3B). Bim is activated by a wide range of apoptotic stimuli in a FOXO3-dependent manner^47^,^48^. Consistent with these observations, enhanced nuclear localization of FOXO3 was observed in Tregs from SYR50-treated middle-aged mice (Fig. 3C and D). These results suggest that SYR50 may limit the frequency of Treg cells through regulation of their apoptosis.

### SYR affects gut microbiota diversity and composition in middle-aged mice

**Figure 4:**
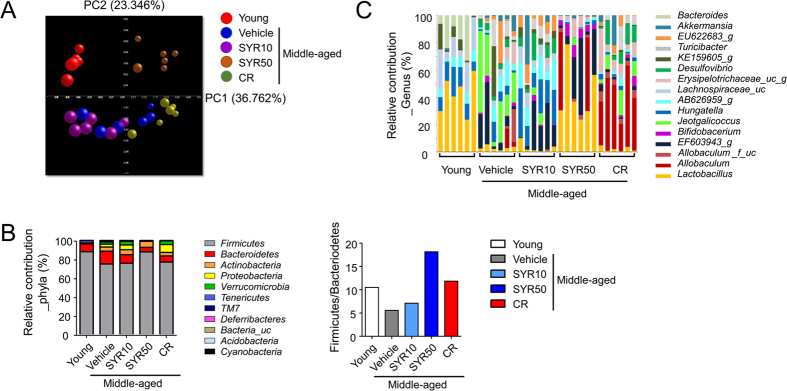
Changes in the gut microbiota due to SYR and CR treatment in middle-aged mice. Middle-aged mice (*n* = 6 each group) were treated with vehicle (control), 10 mg/kg SYR (SYR10), 50 mg/kg SYR (SYR50), or CR for 10 weeks and the gut microbiota of their cecal contents were analyzed by pyrosequencing. (**A**) Bacterial communities were clustered using principal coordinate analysis (PCoA) of weighted UniFrac distance matrices. The first two principal coordinates (PC1 and PC2) from the PCoA of weighted UniFrac are plotted for each sample. The percentage variation in the plotted principal coordinates is indicated on the axes. Each spot represents one sample and each group of mice is denoted by a different color. The size of each dot indicates the diversity index value. (**B**) Microbiota composition at the phylum level (up) and *Firmicutes*/*Bacteroidetes* ratio (down) are shown. Results are expressed as the means ± SEM relative abundance of each phylum. (**C**) The relative microbiota composition (% of total 16S rDNA) at the genus level. Experiments were repeated three times. Data are the averages of three independent experiments.

The gut microbiota plays a critical role in the immune system. Recently, aging has been shown to affect gut microbial communities, which are significantly shaped by diet^20^,^49^. Indeed, life-long CR in mice was shown to establish a structurally balanced architecture of gut microbiota that may contribute to reduction of antigen load from the gut^24^. Based on the protective effects of SYR against immunosenescence (Figs 1, 2 and 3), we examined whether SYR modulates age-associated changes in the gut microbiota. We first compared the overall structural changes of the gut microbiota in young and middle-aged mice treated with vehicle, CR, or SYR by bar-coded pyrosequencing of the V3 region of the 16S rRNA gene. A total of 127495 valid reads from 30 samples were analyzed after the performance of a quality check and normalized to an average of 3600 reads per sample for comparison of diversity indices (Table S4). Shannon diversity indices of the gut microbiota in SYR50-treated middle-aged mice were lower than those of age-matched untreated controls, suggesting that SYR50 treatment may reduce species richness compared with that in control aged mice (Table S4). The data matrix of the weighted UniFrac distance, unweighted pair-group method using arithmetic averages, and principal coordinate analysis (PCoA) showed clear separation among the young mouse groups, SYR50-, CR-, and vehicle (control)-treated middle-aged mouse groups (Fig. 4A)^50^. These results suggest that CR or SYR50 treatment can induce changes in the gut microbiota in middle-aged mice.

Taxonomic assignments of the sequences showed that the *Firmicutes*/*Bacteroidetes* ratio was lower in middle-aged mice than in control young mice (Fig. 4B), consistent with previous reports^24^,^51^,^52^. In SYR-treated middle-aged mouse groups, the *Firmicutes*/*Bacteroidetes* ratio was increased compared with that in age-matched control mouse groups (Fig. 4B). The increase in the phylum *Firmicutes* in SYR-treated mice was due largely to the increase in the genus *Lactobacillus* (38.3% ± 7.8% versus 2.13% ± 0.46% in SYR50 versus age-matched control mice, respectively; *p* = 0.0126) (Figs 4C, S2, and Table S5). In middle-aged mice subjected to CR, a significant increase in the genus *Allobaculum* (35.7% ± 5.6% versus 2.01% ± 1.55% in CR versus control old mice, respectively; *p* = 0.0478) contributed to the increased abundance of the phylum *Firmicutes* (Figs 4C, S2, and Table S5).

**Figure 5:**
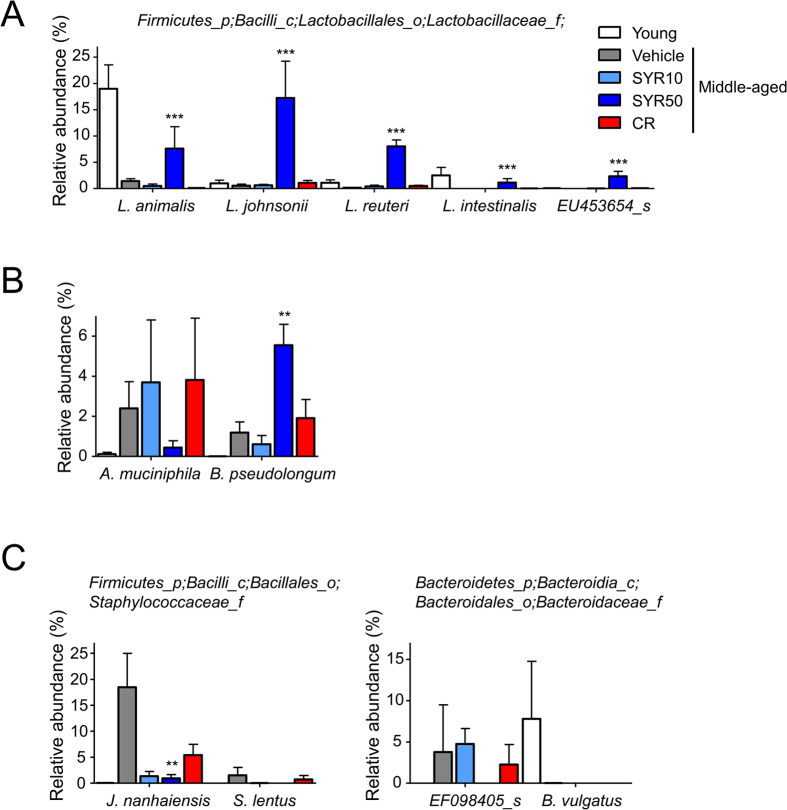
Relative abundance of bacterial species in middle-aged mice subjected to SYR or CR. Middle-aged mice were treated with vehicle (control), 10 mg/kg SYR (SYR10), 50 mg/kg SYR (SYR50), or CR for 10 weeks. Young mice were used as age-mismatched controls. (**A**) Relative abundance of *Lactobacillus* species in cecal contents of old mice supplemented with or without SYR and CR for 10 weeks, and of young mice. (**B**) Relative abundance of the *Bifidobacteriaceae* and *Akkermansia* families. (**C**) Relative abundance of the *Staphylococcaceae* and *Bacteroidaceae* families. Results are expressed as means ± SEM. Statistical analyses were performed using the Kruskal–Wallis test followed by the Mann–Whitney U test. ***P < 0.001, **P < 0.01 versus the untreated middle-aged control group. Experiments were repeated three times. Data are the averages of three independent experiments.

At the species level, SYR50 treatment markedly increased the mean relative abundance of several *Lactobacillus* species, such as *Lactobacillus animalis* (+439%), *Lactobacillus johnsonii* (+3191%), *Lactobacillus reuteri* (+5151%), and *Lactobacillus intestinalis* (+24421%), compared with that of untreated age-matched controls (Fig. 5A). The relative abundance of *Bifidobacterium pseudolongum* (+366%) was also increased in high-dose SYR-treated mice (Fig. 5B). In contrast, the relative abundances of members of the potentially opportunistic pathogenic genus, *Staphylococcaceae* [*Jeotgalicoccus nanhaiensis* (−95%), *Staphylococcus lentus* (−100%), *Bacteroidaceae* (EF098405_s (−100%), and *Bacteroides vulgatus* (−100%)] were negatively affected by SYR50 (Fig. 5C). The relative abundance of *Akkermansia muciniphila* was also decreased by SYR50 treatment (Fig. 5B).

**Figure 6:**
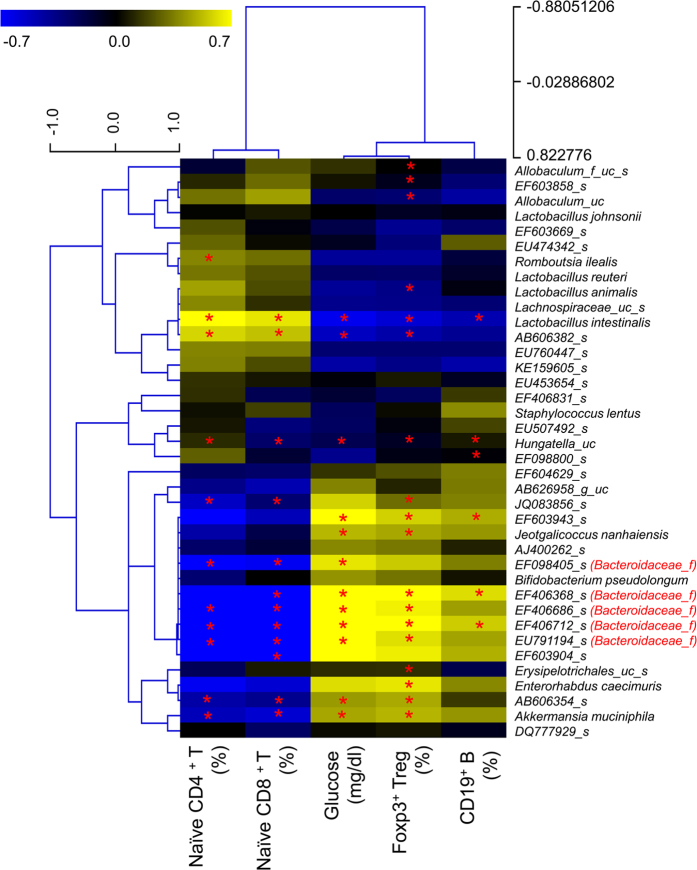
Correlation between the relative abundance of selected bacterial species (prevalence≥10%) and lymphocyte subset frequencies in middle-aged mice subjected to SYR or CR. The heat map shows the nonparametric Kendall’s tau rank-correlation coefficients between bacterial abundance and lymphocyte subset frequencies and blood glucose levels. *P < 0.05. Experiments were repeated three times. Data are the averages of three independent experiments.

Next, we used the Kendal tau rank-correlation coefficient to measure directly the correlation between gut microbiota phylotypes and the proportions of lymphocyte subpopulations (Fig. 6). Five operational taxonomic units (OTUs) in *Bacteroidaceae* were positively correlated with Treg cell frequencies and also with blood glucose levels, but negatively correlated with the frequencies of naïve CD4^+^ and CD8^+^ T cells. *Akkermansia muciniphila* showed a positive correlation with blood glucose levels and a negative correlation with frequencies of naïve CD4^+^ and CD8^+^ T cells. *Jeotgalicoccus nanhaiensis* showed a positive correlation with blood glucose levels and frequencies of Treg and B cells, and a negative correlation with frequencies of naïve CD4^+^ and CD8^+^ T cells, whereas *Lactobacillus intestinalis* was negatively correlated with frequencies of Treg and B cells and blood glucose levels but positively correlated with frequencies of naïve CD4^+^ and CD8^+^ T cells.

**Figure 7:**
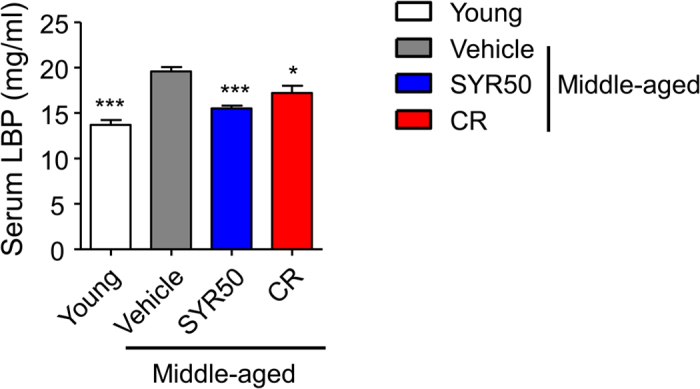
Effects of SYR and CR on the levels of LBP, a gut-derived antigen load marker. Middle-aged mice were treated with vehicle (control), 50 mg/kg SYR (SYR50), or CR for 10 weeks. Young mice were used as age-mismatched controls. LBP levels were measured in serum as described in the Materials and Methods. Experiments were repeated three times. Data are the averages of three independent experiments. All data are expressed as means ± SEM. Statistical analyses were performed using the Kruskal-Wallis test followed by Mann-Whitney U test. ***P < 0.001, *P < 0.05 versus the untreated middle-aged control group.

In addition, to determine whether the SYR- or CR-induced changes in the composition of the gut microbiota were associated with reduced antigen load in middle-aged mice, we measured the serum levels of lipopolysaccharide (LPS)-binding protein (LBP)^39^. LBP is considered a marker of the gut-derived antigen load that links the antigen load in blood with the host inflammatory response^53^. Middle-aged mice treated with SYR50 and CR exhibited significantly reduced LBP levels in serum compared with untreated age-matched control mice (Fig. 7), suggesting a protective role of SYR50 in gut permeability. Taken together, these findings suggest that the administration of SYR50 could positively modulate gut integrity as well as microbiota diversity and composition in middle-aged mice^24^,^54^.

### SYR induces enhanced antibody responses to influenza vaccination

**Figure 8:**
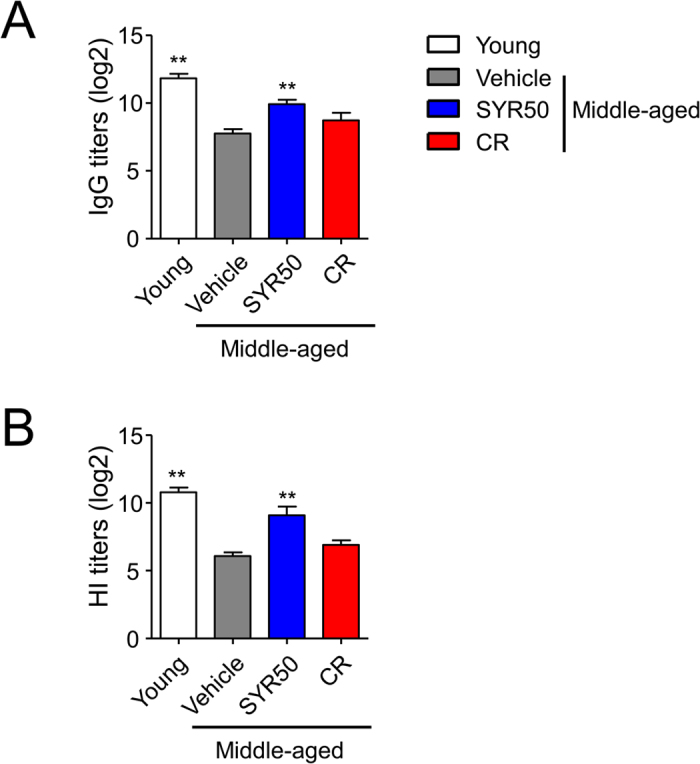
Effects of SYR50 and CR on the humoral immune response to influenza vaccination. Middle-aged mice were treated with vehicle (control), 50 mg/kg SYR (SYR50), or CR for 10 weeks and bled 2 weeks after the second immunization. Young mice were used as age-mismatched controls. Influenza-specific antibody titers (**A**) and hemagglutination inhibition (HI) titers (**B**) were determined. Values are means ± SEM, *n* = 6. Statistical analyses were performed using the Kruskal–Wallis test followed by the Mann–Whitney U-test. **P < 0.01 versus the untreated middle-aged control group. Experiments were repeated three times. Data are the averages of three independent experiments.

As SYR increased the frequencies of total CD3^+^ T cells and naïve T cells in both CD4^+^ and CD8^+^ T-cell populations (Fig. 1) and their functions *in vitro* upon TCR stimulation (Fig. 2), we further examined whether SYR50 could enhance humoral immune responses to influenza vaccine *in vivo* by measuring anti-influenza HA antibody titers following influenza vaccination. Middle-aged mice were treated with vehicle (control), SYR50, or CR for 10 weeks and subcutaneously immunized twice at 3-week intervals. Two weeks after the final vaccination, HA-specific IgG titers and HA inhibition (HI) assay were performed. SYR50-treated mice showed a significant increase in HA-specific IgG titer compared with untreated age-matched control mice (Fig. 8A), whereas CR had no effect. As HA inhibition (HI) assay is used to measure protective (neutralizing) antibody levels produced during the primary B-cell response postvaccination^55^, we also measured HI antibody titers in mice immunized with influenza vaccine (Fig. 8B). SYR50 treatment significantly increased HI titers compared with those in untreated age-matched control mice as well as middle-aged mice subjected to CR. Taken together, these findings suggest that SYR-induced enhancement of cellular immunity (Figs 1, 2 and 3) also augments humoral immunity in response to influenza vaccination in the middle-aged host.

## Discussion

Immunosenescence and related health problems include enhanced susceptibility to infectious diseases, autoimmunity, cancer, and decreased responsiveness to vaccination^3^,^4^,^5^. Given the acceleration of population aging and the concomitant increases in rates of age-related diseases and disabilities, prevention or retardation of age-related immunological dysfunction would be important to extending the healthy lifespan of individuals. In this study, we examined whether syringaresinol (SYR), a polyphenolic chemical substance isolated from *Panax ginseng* berry pulp, could modulate immunosenescence using a middle-aged mouse model, representing the human ages of 45–65 as defined by the standard diagnostic manual of the American Psychiatric Association. The T-cell subset profile is known to be a predictor of longevity, which helps reduce disease risk later in life^36^,^56^. We demonstrated that high-dose SYR (SYR50) treatment delayed the age-related alterations in naïve T-cell and Treg-cell populations and reduced inflammaging. SYR50 treatment significantly enhanced the population of beneficial *Lactobacillus* and *Bifidobacterium* bacteria, while reducing opportunistic pathogens. SYR50 also enhanced humoral immunity against influenza vaccination to the level of young healthy controls. Thus, SYR may contribute to the reversal of immunosenescence in middle-aged mice through the modulation of gut integrity as well as the microbiota diversity and composition.

The elderly frequently suffer from severe infections due to age-related immune dysfunction. Recent studies have shown that marked increases in Foxp3^+^ Treg cells with aging in both mice and humans increase the risk of infectious diseases in the elderly^57^. This age-related accumulation of Foxp3^+^ Treg cells is due to enhanced survival of Tregs rather than enhanced *in vivo* proliferation^46^ in which Bim may play a central role in regulating Treg lifespan. Bim is known to be activated by a wide range of apoptotic stimuli at the transcriptional level in a FOXO3-dependent manner^47^,^48^. FOXO ensures the survival of maximal numbers of naïve T cells^33^, modulates helper T-cell quiescence^34^, and prevents inflammation-induced apoptosis of neutrophils^35^ that contributes to intestinal resistance against infection and preservation of the microbiota^58^. FOXO plays a key role in T-cell homeostasis and regulation of inflammation and immunity^59^,^60^,^61^. The observation that SYR50 increased the nuclear localization of FOXO3 in Treg cells (Fig. 3) suggests that increased expression of Bim via FOXO3 activation is a potential mechanism for the protective effect of SYR against age-related increase in the Treg population through increased expression of Bim via FOXO3 activation. Further studies are needed to delineate the precise molecular mechanism underlying the effect of SYR50 in enhancing nuclear translocation of FOXO3, but such detailed investigations are beyond the scope of the present study.

Reducing chronic antigenic load has been reported to counteract immunosenescence^13^. Alteration of the gut microbiota composition with aging may lead to continuous antigenic stimulation and consequently to immunosenescence^20^,^49^,^62^. Therefore, reconstitution of a normal or healthy microbiota in the elderly has been proposed as an anti-immunosenescence intervention^15^,^63^. However, little is known about the distinct gut microbiota structure that reverses immunosenescence. SYR50 modified the age-related alteration in the gut microbiota structure (Figs 4, 5 and 6); for example, it increased the *Firmicutes*/*Bacteroidetes* ratio, increased the abundance of the beneficial genera *Lactobacillus* and *Bifidobacterium*, and decreased the abundance of opportunistic pathogenic *Staphylococcaceae*^24^ and *Bacteroidaceae*^64^, which may increase the efficacy of vaccines^65^. Indeed, in the elderly, a significant reduction in the *Firmicutes*/*Bacteroidetes* ratio and an increase in facultative anaerobes, such as *Streptococcus* and *Staphylococcus*, were associated with increased levels of inflammatory cytokines^66^. In particular, *Bifidobacterium* was found in all centenarians, whereas it was not detected in the elderly^66^,^67^. Furthermore, Ouwehand *et al*.^22^ reported that the consumption of probiotic *Bifidobacterium* resulted in increased levels of various intestinal *Bifidobacterium* species accompanied by decreases in IL-10 and TNF-α levels in the elderly, suggesting that modulation of the intestinal *Bifidobacterium* microbiota could reduce the age-dependent inflammatory status. SYR50 treatment increased phylotypes that were positively correlated with naïve T-cell frequency but negatively correlated with Treg frequency (Figs 5 and 6). Among the bacteria enriched in SYR-treated middle-aged mice, *Lactobacillus rhamnosus* GG has been shown to reduce the antigen load to the host and alleviate inflammation^68^. Oral administration of the probiotic *Lactobacillus casei* Shirota improved the immune response to influenza virus infection in aged mice^69^. These results suggest that alteration of gut microbiota diversity and composition induced by SYR50 treatment in middle-aged mice is closely related to enhanced immune responses to influenza vaccination (Fig. 8).

A surprising aspect of our study was the contrasting effect of CR on the immune system of middle-aged mice. CR was found to maintain several age-sensitive immune parameters, including the frequency of naïve T cells and the proliferative capacity of T cells, and to inhibit age-related dysregulation of cytokine production. However, CR did not improve the impaired humoral immune response to influenza vaccination in middle-aged mice (Fig. 8). Previous studies have shown that Treg cells inhibit protective immune responses to viruses and vaccines, and depletion of Treg cells significantly enhances the immunogenicity of vaccines^70^,^71^. Based on these reports, the failure of CR to reduce Treg frequency with aging (Fig. 1) may inhibit the humoral immune response to influenza vaccination. Our data were consistent with previous reports that CR decreased the antibody responses to vaccination and then increased mortality following inoculation with live influenza virus^41^,^42^. In addition, alteration of gut microbiota composition may play a role in the ensuing immune response. Compared with mice subjected to SYR50, CR showed higher abundance of *Proteobacteria*–*Desulfovibrio* but lower levels of *Firmicutes*–*Lactobacillus* and *Actinobacteria*–*Bifidobacterium*. In general, the abundance of the phylum *Proteobacteria*, which includes a wide variety of pathogens, is elevated in the aged gut, and their load has therefore been suggested as a potential diagnostic criterion for dysbiosis^66^,^72^. Unlike SYR50 treatment, the gut microbiota of mice subjected to CR had fewer bacteria that directly downregulate proinflammatory responses, which may contribute to the deleterious effects of CR on vaccine efficacy. Our findings regarding the bacterial communities of mice subjected to CR were in contrast to previous reports of the increased abundance of the genera *Lactobacillus* and *Bifidobacterium* after CR treatment for more than 57 weeks^24^. These discrepant findings regarding the CR-induced gut microbiota composition may have been due to differences in the duration of CR treatment. As the status of the gut microbiota is a critical determinant of vaccine efficacy^73^, we cannot exclude the possibility that long-term CR prevents the age-dependent decline in the immune response to vaccination. Although our data collectively suggest that SYR-mediated immune modulation in middle aged mice is closely associated with an alteration of gut microbiota, more detailed and precise examination on the underlying mechanism is required. In the future, we will test whether administration of antibiotics could abolish the effect of SYR on immune and endocrine system in the SPF mice. In addition, given the fact that SIRT1 and FOXO3 participate in the regulation of glucose metabolism and Treg homeostasis^46^,^74^,^75^, and SYR induces the activation of SIRT1 and FOXO3^31^ (Fig. 3), still there is a possibility that SYR may have a direct effect on the level of blood glucose as well as Treg homeostasis. Comparing the effect of SYR treatment in the conventional SPF and germ free mice will clarify whether SYR directly modulates host immune system and endocrine system, or indirectly works through an alteration of gut microbiota, or both mechanisms are equally involved.

In summary, our findings indicated that administration of SYR50 effectively improved the age-related dysregulation of lymphocyte subsets. SYR50 treatment restored naïve T-cell frequencies, properly regulated Treg-cell homeostasis *via* upregulation of Bim expression, and lowered the antigen load through reconstitution of a healthy gut microbiota enriched in *Lactobacillus* and *Bifidobacterium*. Although further studies are necessary to elucidate the mechanisms by which SYR modulates the gut microbial population and its subsequent impact on the immune system, our data suggest that modulation of the gut microbiota composition and improvement of age-altered Treg homeostasis may facilitate development of anti-immunosenescence interventions.

## Materials and Methods

### Animals

Young (6 weeks old) and middle-aged (40 weeks old) male C57BL/6 mice and ICR mice (male and female, 8 weeks old, for toxicity studies) were purchased from Central Laboratory Animal Inc. (Seoul, Republic of Korea). Male Sprague–Dawley rats (SD rats, 3 months old, for pharmacokinetics analysis) were purchased from Samtako Inc. (Osan, Republic of Korea). Animals were allowed to acclimatize to their surroundings for at least 2 weeks before the experiments. Animals were housed in sterile cages under specific pathogen-free conditions in the animal facility of PACIFICPHARMA Corporation (Giheung, Republic of Korea). All experimental procedures were performed in accordance with National Institutes of Health (NIH) Guidelines for the Care and Use of Laboratory Animals, and were approved by Institutional Animal Care and Use Committee (IACUC) of PACIFICPHARMA Corporation (approval #IACUC14–014). Animals were maintained in accordance with the National Animal Welfare Law of Republic of Korea. At 44 weeks of age, mice were randomly assigned to one of the following four groups (*n* = 12) and kept in individual cages: (1) normal diet with vehicle as age-matched controls; (2) normal diet with 10 mg/kg syringaresinol (SYR10); (3) normal diet with 50 mg/kg SYR (SYR50); and (4) caloric restriction (CR) of normal diet by 30%. Young (10 weeks old) mice were used as age-mismatched controls. SYR was synthesized by Hanchem (http://www.hanchem.net). The purity of the compound was >99.7%. SYR was administered at 10 or 50 mg/kg body weight in 1% methylcellulose using an oral zonde needle once daily for 10 weeks. CR was initiated at 42 weeks old by restricting food intake by 10% compared with that of *ad libitum* controls, progressing to 20% at 43 weeks and 30% at 44 weeks; the final level (Table S1, 2.4 g daily, D12450B; Research Diets, New Brunswick, NJ, USA) was maintained for 10 weeks. Body weight, food intake, and blood glucose concentration were measured at the indicated time points.

### Pharmacokinetics and toxicity of SYR

SYR was administered intravenously (i.v., 1 mg/kg) to SD rats. The oral dose of SYR was dispersed in 1% methylcellulose to final concentrations of 2 and 20 mg/kg. Blood samples were collected from the retroorbital plexus before (0 h) and at several time points (10, 20, 30 min, 1, 2, 3, and 5 h for i.v. and 10, 20, 30 min, 1, 2, 3, 5, 7, 24, and 48 h for oral administration) after dosing. Plasma SYR concentration was determined using a UPLC-MS/MS system (Table S6).

All toxicity studies were performed according to the OECD guidelines for testing of chemicals; Test No. 420 Acute Oral Toxicity. SYR was orally administered once to a group of five male and five female ICR mice, following an overnight fast, at a dose of 2 g/kg to evaluate its toxicity. Mortality, clinical signs, body weight, and gross necropsy findings were evaluated. No changes in general condition, autopsy examination results, or blood biochemical tests due to drug administration were observed. All animals showed constant body weight gain over the 14-day observation period. Based on the results of this study, the minimal lethal dose (MLD) for SYR administered orally to ICR mice was determined to be >2 g/kg.

### Flow cytometric analysis

Single-cell suspensions (0.5–1 × 10^6^ cells in PBS containing 2% fetal bovine serum) from the spleen were incubated with purified anti-CD16/32 antibodies (Abs) (FcγRII/III block, 2.4G2; eBioscience, San Diego, CA, USA) to block nonspecific staining. Splenic T-cell subsets were identified by immunostaining with fluorochrome-conjugated Abs to CD3 (eBioscience), CD4 (eBioscience), CD8 (BD Biosciences, San Jose, CA, USA), CD44 (BD Biosciences), and CD62L (BioLegend, San Diego, CA, USA). Intracellular foxp3 and Bim staining was performed using Abs to foxp3 (eBioscience) or Bim (Cell Signaling Technology, Danvers, MA, USA). For splenic B cell subsets, cell staining included Abs to CD19, CD21, and CD23 (all from BD Biosciences). Cells were stained with Abs against CD11c (BioLegend), CD11b (BD Biosciences), Ly6C (eBioscience), or Ly6G (eBioscience) for dendritic cells, monocytes, and granulocytes, respectively. Data were acquired on an LSRII flow cytometer (BD Biosciences) and analyzed using FlowJo software (ver. 9.7.6, TreeStar, Ashland, OR, USA).

Cell counts were performed in duplicate after addition of Trypan blue dye using a Vi-CELL Series Cell Viability Analyzer (Beckman Coulter, Brea, CA, USA).

### Immunofluorescence staining

CD4^+^CD25^+^ regulatory T (Treg) cells were enriched from splenocytes using a Mouse Treg Isolation Kit (Miltenyi Biotec, Bergisch Gladbach, Germany) and then subjected to the following procedure. Tregs were fixed with 3% formaldehyde in PBS, permeabilized with 70% ethanol, and incubated with anti-FOXO3 (FOXO3a; Cell Signaling Technology) antisera in microscopy buffer (2% BSA and 0.1% Triton X-100 in PBS), followed by Alexa 488-conjugated donkey anti-rabbit IgG (Molecular Probes™, Thermo Fisher Scientific, Fremont, CA, USA). DNA was stained with 4′,6-diamidino-2-phenylindole (DAPI; Thermo Fisher Scientific). Specimens were observed using a Zeiss Axioplan-2 microscope (Oberkochen, Germany). Image acquisition and post-processing were performed using the Northern Eclipse software (Empix, Mississauga, ON, Canada).

### Subcellular fractionation

Tregs were washed three times with ice-cold PBS and scraped into homogenization buffer (50 mM HEPES, pH 7.4, 255 mM sucrose, 1 mM EDTA) containing a protease inhibitor mixture (Sigma). After homogenization with 20 strokes in a glass homogenizer, the homogenate was centrifuged at 1000 × *g* for 10 minutes into supernatant 1 and pellet. The pellet was homogenized in 10 mM Tris buffer (pH 7.5) containing 300 mM sucrose, 1 mM EDTA, and protease inhibitor mixture, and centrifuged at 5000 × *g* for 5 minutes. The resulting pellet was the nuclear fraction. Supernatant 1 was removed and centrifuged at 5000 × *g* for 20 minutes to yield the pellet (mitochondrial fraction) and supernatant 2 (cytosolic fraction).

### Immunoblotting

Cells were lysed in RIPA buffer (PBS, pH 7.4, containing 1% NP-40, 0.5% sodium deoxycholate, 0.1% SDS) with protease inhibitor cocktail (Sigma-Aldrich, Yongin, Republic of Korea). Aliquots of 40 μg of protein were resolved on 4–12% NuPAGE gels run in an MES buffer system (Invitrogen, Gaithersburg, MD, USA) and transferred onto PVDF membranes according to the manufacturer’s protocol. Immunoreactive proteins were revealed by enhanced chemiluminescence with ECL Plus (Amersham, GE Healthcare, Little Chalfont, UK). Antibodies against Lamin B, FOXO3, and cyclophilin A were purchased from Santa Cruz Biotechnology (Santa Cruz, CA, USA), Cell Signaling Technologies (Danvers, MA, USA), and Upstate Biotechnology (Lake Placid, NY, USA), respectively.

### Activation of splenic T cells

Splenocytes (1 × 10^6^ per well) were incubated with soluble anti-CD3 (1 μg/mL; eBioscience) and anti-CD28 (1 μg/mL; eBioscience) Abs or IgG (BD Biosciences) for 3 days. Incorporation of ^3^H-thymidine (1 μCi/well; Perkin Elmer, Waltham, MA, USA) by proliferating cells was measured during the last 18 hours of culture. The culture supernatants were analyzed for cytokine content (IL-2 and IFN-γ) using the BioPlex kit mouse-specific cytokine standards (Bio-Rad, Hercules, CA, USA).

### Immunization

Influenza vaccine was prepared using the hemagglutinin protein (HA) of A/Puerto Rico/8/34 (H1N1) influenza virus (Sino Biological Inc., Beijing, China). The untreated middle-aged control group, SYR50 group, CR group, and young group (*n* = 6 per group) were subcutaneously immunized twice at 3-week intervals with a 1:1 mixture of vaccine (0.3 μg/ml per subunit) and adjuvant (Stimune; Cedi-Diagnostics, Lelystad, The Netherlands) in a total volume of 100 μL. The negative control group received concurrent injections with a 1:1 mixture of PBS and adjuvant in a total volume of 100 μL.

### Measurement of HA-specific antibody levels

Two weeks after the final vaccination, anti-influenza HA-specific Abs in serum samples were measured by indirect enzyme-linked immunosorbent assay using 96-well plates coated with HA (0.1 μg) in 0.1 M bicarbonate buffer (pH 9.6) overnight at 4 °C and blocked with 5% BSA in PBS overnight at 4 °C. Mouse serum (1/100 dilution in 1% BSA/PBS) was added to plates and incubated for 2 hours at 37 °C, washed, and then incubated with anti-mouse IgG (H + L) Ab-Biotin conjugate (Abcam, Cambridge, MA) for 2 hours at room temperature. Plates were washed, and bound anti-IgG Ab-Biotin conjugates were revealed by adding streptavidin-HRP conjugate (Jackson ImmunoResearch, West Grove, PA, USA), and developed in 3,3′,5,5′-tetramethylbenzidine (TMB) substrate (BD Biosciences). The optical density at 450 nm was read using an ELISA plate reader (Molecular Devices, Sunnyvale, CA, USA). Values above the cut-off background level (mean value of sera from saline-immunized mice multiplied by a factor of 2.1) were considered positive. Titers are shown as reciprocal end-dilutions.

### Hemagglutination inhibition (HI) assay

Serum HI titers were measured using the standard protocol described previously^76^. Briefly, to eliminate nonspecific inhibitors, serum was treated overnight with receptor-destroying enzyme (RDE; Sigma-Aldrich, St. Louis, MO, USA). Serial twofold dilutions of serum (25 μL) were mixed with 4 HA units of influenza virus A/PR/8/34 and incubated at room temperature for 30 minutes, followed by addition of 1% chicken red blood cells. The mixture was further incubated for 60 minutes. The HI titer was determined as the reciprocal of the lowest dilution that showed inhibition of HA.

### Gut microbiota analysis

Serum lipopolysaccharide (LPS)-binding protein (LBP) levels were determined after 1:800 dilution of samples using a Mouse LBPELISA kit (Cell Sciences, Canton, MA, USA) according to the manufacturer’s protocol.

Ceca contents were collected from the mice in the above groups, immediately frozen in liquid nitrogen, and stored at −80 °C until analysis. DNA was extracted using a FastDNA SPIN extraction kit (MP Biomedicals, Santa Ana, CA, USA). PCR amplification of the V1–V3 region of bacterial 16S rRNA gene was performed using universal primers incorporating the FLX Titanium adapters and a sample barcode sequence. The forward primer (27F, bold type) was 5′-CCT-ATC-CCC-TGT-GTG-CCT-TGG-CAG-TC*T-CAG*-Linker-**AGA-GTT-TGA-TCC-TGG-CTC-AG**-3′, in which the sequence of the 454 adapter 2 is shown underlined and the 454 key in italics. The reverse primer (536R, bold type) was 5′-CCA-TCT-CAT-CCC-TGC-GTG-TCT-CCG-AC*T-C*-AG-Barcode-Linker-**TTA-CCC-GCG-GCT-GCT-GGC-AC**-3′, in which the 454 adapter 1 sequence is underlined and the 454 key is shown in italics. The amplified PCR products were purified, quantified, and subjected to emulsion PCR using protocols developed by Roche Life Sciences (Roche, Branford, CT). Pyrosequencing was performed by ChunLab Inc. (Seoul, Korea) using a 454b GS FLX Titanium Sequencing System according to the method described by Chun *et al*.^77^. A total of 454 pyrosequencing reads have been deposited in the NCBI short read archive (accession number GSE75825).

Sequences were sorted using their unique barcode, and low-quality reads (average quality score <25 or read length <300 bp) were removed. Primer sequences were trimmed by pairwise alignment and with the hmm-search program of the HMMER 3.0 package^78^. Trimmed sequences were clustered to correct for sequencing errors, and representative sequences were assigned to operational taxonomic units (OTUs; 97% identity). Taxonomic identification was performed using the EzTaxon-e database (http://eztaxon-e.ezbiocloud.net/) by the highest pairwise similarity among the top five BLASTN results^79^. Possible chimeric sequences were removed using the UCHIME software^79^. The read numbers in each sample were normalized by random subsampling, and the diversity indices were calculated using Mothur software^80^. UniFrac clustering, heat map, and principal coordinate analyses were performed using CLcommunity (ChunLab). We also performed LEfSe analysis on the website http://huttenhowe.sph.harvard.edu/galaxy.

### Statistical analysis

Results are presented as means ± standard error of the mean (SEM). Nonparametric methods used SPSS version 20 (IBM SPSS, Armonk, NY) because of the low sample size (*n* = 6). On the basis of the relative abundance analysis using LEfSe and the results of the Kruskal–Wallis and Mann–Whitney U tests, *P* < 0.05 was taken to indicate significance, and the threshold logarithmic linear discriminant analysis (LDA) score was 4.0. The nonparametric Kendall tau rank-correlation matrixes between gut microbiota composition and lymphocyte subset frequencies were generated to create a heat map using the Multiple Experimental Viewer software (MEV; v. 4.8.1). Frequencies and numbers of immune cells, T cell proliferation and cytokine assay data were analyzed by one-way ANOVA (followed by Dunnett’s post hoc test).

## Additional Information

**How to cite this article**: Cho, S.-Y. *et al*. Modulation of gut microbiota and delayed immunosenescence as a result of syringaresinol consumption in middle-aged mice. *Sci. Rep.* **6**, 39026; doi: 10.1038/srep39026 (2016).

**Publisher's note:** Springer Nature remains neutral with regard to jurisdictional claims in published maps and institutional affiliations.

## Supplementary Material

Supplementary Information

## References

[b1] Kennedy B. K. . Geroscience: linking aging to chronic disease. Cell 159, 709–713, doi: 10.1016/j.cell.2014.10.039 (2014). 25417146 PMC4852871

[b2] Franceschi C. & Campisi J. Chronic Inflammation (Inflammaging) and Its Potential Contribution to Age-Associated Diseases. Journals of Gerontology Series a-Biological Sciences and Medical Sciences 69, S4–S9, doi: 10.1093/gerona/glu057 (2014). 24833586

[b3] Fulop T. . Immunosenescence and cancer. Crit Rev Oncog 18, 489–513 (2013). 24579731 10.1615/critrevoncog.2013010597

[b4] Krupica T. Jr., Fry T. J. & Mackall C. L. Autoimmunity during lymphopenia: a two-hit model. Clin Immunol 120, 121–128, doi: 10.1016/j.clim.2006.04.569 (2006). 16766227

[b5] Goronzy J. J. & Weyand C. M. Understanding immunosenescence to improve responses to vaccines. Nat Immunol 14, 428–436, doi: 10.1038/ni.2588 (2013). 23598398 PMC4183346

[b6] Gavazzi G. & Krause K. H. Ageing and infection. Lancet Infect Dis 2, 659–666 (2002). 12409046 10.1016/s1473-3099(02)00437-1

[b7] Goronzy J. J., Fang F., Cavanagh M. M., Qi Q. & Weyand C. M. Naive T cell maintenance and function in human aging. J Immunol 194, 4073–4080, doi: 10.4049/jimmunol.1500046 (2015). 25888703 PMC4452284

[b8] Garg S. K. . Aging is associated with increased regulatory T-cell function. Aging Cell 13, 441–448, doi: 10.1111/acel.12191 (2014). 24325345 PMC4032602

[b9] Frasca D. & Blomberg B. B. Effects of aging on B cell function. Curr Opin Immunol 21, 425–430, doi: 10.1016/j.coi.2009.06.001 (2009). 19608393 PMC2853364

[b10] Pinchuk L. M. & Filipov N. M. Differential effects of age on circulating and splenic leukocyte populations in C57BL/6 and BALB/c male mice. Immun Ageing 5, 1, doi: 10.1186/1742-4933-5-1 (2008). 18267021 PMC2268915

[b11] Weiskopf D., Weinberger B. & Grubeck-Loebenstein B. The aging of the immune system. Transpl Int 22, 1041–1050, doi: 10.1111/j.1432-2277.2009.00927.x (2009). 19624493

[b12] Nikolich-Zugich J. & Messaoudi I. Mice and flies and monkeys too: caloric restriction rejuvenates the aging immune system of non-human primates. Exp Gerontol 40, 884–893, doi: 10.1016/j.exger.2005.06.007 (2005). 16087306

[b13] Capri M. . Complexity of anti-immunosenescence strategies in humans. Artif Organs 30, 730–742, doi: 10.1111/j.1525-1594.2006.00295.x (2006). 17026572

[b14] Adolfsson O., Huber B. T. & Meydani S. N. Vitamin E-enhanced IL-2 production in old mice: naive but not memory T cells show increased cell division cycling and IL-2-producing capacity. J Immunol 167, 3809–3817 (2001). 11564798 10.4049/jimmunol.167.7.3809

[b15] Candore G. . Immunosenescence and anti-immunosenescence therapies: the case of probiotics. Rejuvenation Res 11, 425–432, doi: 10.1089/rej.2008.0662 (2008). 18442326

[b16] Messaoudi I. . Delay of T cell senescence by caloric restriction in aged long-lived nonhuman primates. Proc Natl Acad Sci USA 103, 19448–19453, doi: 10.1073/pnas.0606661103 (2006). 17159149 PMC1748246

[b17] Pahlavani M. A. Caloric restriction and immunosenescence: a current perspective. Front Biosci 5, D580–587 (2000). 10833464 10.2741/pahlavani

[b18] Spaulding C. C., Walford R. L. & Effros R. B. Calorie restriction inhibits the age-related dysregulation of the cytokines TNF-alpha and IL-6 in C3B10RF1 mice. Mech Ageing Dev 93, 87–94 (1997). 9089573 10.1016/s0047-6374(96)01824-6

[b19] Fontana L. & Partridge L. Promoting health and longevity through diet: from model organisms to humans. Cell 161, 106–118, doi: 10.1016/j.cell.2015.02.020 (2015). 25815989 PMC4547605

[b20] Biagi E. . Ageing and gut microbes: perspectives for health maintenance and longevity. Pharmacol Res 69, 11–20, doi: 10.1016/j.phrs.2012.10.005 (2013). 23079287

[b21] Tien M. T. . Anti-inflammatory effect of Lactobacillus casei on Shigella-infected human intestinal epithelial cells. J Immunol 176, 1228–1237 (2006). 16394013 10.4049/jimmunol.176.2.1228

[b22] Ouwehand A. C. . Bifidobacterium microbiota and parameters of immune function in elderly subjects. FEMS Immunol Med Microbiol 53, 18–25, doi: 10.1111/j.1574-695X.2008.00392.x (2008). 18336547

[b23] Ibrahim F. . Probiotics and immunosenescence: cheese as a carrier. FEMS Immunol Med Microbiol 59, 53–59, doi: 10.1111/j.1574-695X.2010.00658.x (2010). 20236323

[b24] Zhang C. . Structural modulation of gut microbiota in life-long calorie-restricted mice. Nat Commun 4, 2163, doi: 10.1038/ncomms3163 (2013). 23860099 PMC3717500

[b25] Smeds A. I., Eklund P. C. & Willfor S. M. Content, composition, and stereochemical characterisation of lignans in berries and seeds. Food Chem 134, 1991–1998, doi: 10.1016/j.foodchem.2012.03.133 (2012). 23442648

[b26] Yamazaki T., Shimosaka S., Sasaki H., Tukiyama T. & Tokiwa T. (+)-Syringaresinol-di-O-beta-D-glucoside, a phenolic compound from Acanthopanax senticosus Harms, suppresses proinflammatory mediators in SW982 human synovial sarcoma cells by inhibiting activating protein-1 and/or nuclear factor-kappaB activities. Toxicol. In Vitro. 21, 1530–1537 (2007). 17561367 10.1016/j.tiv.2007.04.016

[b27] Chung B. H. . Syringaresinol causes vasorelaxation by elevating nitric oxide production through the phosphorylation and dimerization of endothelial nitric oxide synthase. Exp. Mol. Med. 44, 191–201 (2012). 22170035 10.3858/emm.2012.44.3.014 PMC3317483

[b28] Lajter I. . Inhibition of COX-2 and NF-kappaB1 Gene Expression, NO Production, 5-LOX, and COX-1 and COX-2 Enzymes by Extracts and Constituents of Onopordum acanthium. Planta Med 81, 1270–1276, doi: 10.1055/s-0035-1546242 (2015). 26383017

[b29] Heinonen S. . *In vitro* metabolism of plant lignans: new precursors of mammalian lignans enterolactone and enterodiol. J Agric Food Chem 49, 3178–3186 (2001). 11453749 10.1021/jf010038a

[b30] Cho S. . Syringaresinol protects against hypoxia/reoxygenation-induced cardiomyocytes injury and death by destabilization of HIF-1alpha in a FOXO3-dependent mechanism. Oncotarget 6, 43–55 (2015). 25415049 10.18632/oncotarget.2723 PMC4381577

[b31] Cho S. Y., Cho M., Seo D. B., Lee S. J. & Suh Y. Identification of a small molecule activator of SIRT1 gene expression. Aging (Albany NY) 5, 174–182 (2013). 23525956 10.18632/aging.100539 PMC3629289

[b32] van der Horst A. & Burgering B. M. Stressing the role of FoxO proteins in lifespan and disease. Nat Rev Mol Cell Biol 8, 440–450, doi: 10.1038/nrm2190 (2007). 17522590

[b33] Hedrick S. M., Hess Michelini R., Doedens A. L., Goldrath A. W. & Stone E. L. FOXO transcription factors throughout T cell biology. Nat Rev Immunol 12, 649–661, doi: 10.1038/nri3278 (2012). 22918467 PMC3875397

[b34] Kerdiles Y. M. . Foxo transcription factors control regulatory T cell development and function. Immunity 33, 890–904, doi: 10.1016/j.immuni.2010.12.002 (2010). 21167754 PMC3034255

[b35] Peng S. L. Foxo in the immune system. Oncogene 27, 2337–2344, doi: 10.1038/onc.2008.26 (2008). 18391975

[b36] Miller R. A., Chrisp C. & Galecki A. CD4 memory T cell levels predict life span in genetically heterogeneous mice. FASEB J 11, 775–783 (1997). 9271362 10.1096/fasebj.11.10.9271362

[b37] Miller R. A. & Chrisp C. T cell subset patterns that predict resistance to spontaneous lymphoma, mammary adenocarcinoma, and fibrosarcoma in mice. J Immunol 169, 1619–1625 (2002). 12133992 10.4049/jimmunol.169.3.1619

[b38] Rodgers J. T. . Nutrient control of glucose homeostasis through a complex of PGC-1 alpha and SIRT1. Nature 434, 113–118, doi: 10.1038/nature03354 (2005). 15744310

[b39] Sun L. . A marker of endotoxemia is associated with obesity and related metabolic disorders in apparently healthy Chinese. Diabetes Care 33, 1925–1932, doi: 10.2337/dc10-0340 (2010). 20530747 PMC2928335

[b40] Martinet K. Z., Bloquet S. & Bourgeois C. Ageing combines CD4 T cell lymphopenia in secondary lymphoid organs and T cell accumulation in gut associated lymphoid tissue. Immunity & Ageing 11, doi: Artn 810.1186/1742-4933-11-8 (2014). 10.1186/1742-4933-11-8 PMC4020584 24829607

[b41] Gardner E. M. Caloric restriction decreases survival of aged mice in response to primary influenza infection. J Gerontol A Biol Sci Med Sci 60, 688–694 (2005). 15983169 10.1093/gerona/60.6.688

[b42] Goldberg E. L. . Lifespan-extending caloric restriction or mTOR inhibition impair adaptive immunity of old mice by distinct mechanisms. Aging Cell 14, 130–138, doi: 10.1111/acel.12280 (2015). 25424641 PMC4326902

[b43] Wakikawa A., Utsuyama M., Wakabayashi A., Kitagawa M. & Hirokawa K. Age-related alteration of cytokine production profile by T cell subsets in mice: a flow cytometric study. Exp Gerontol 34, 231–242 (1999). 10363789 10.1016/s0531-5565(98)00062-x

[b44] Thoman M. L. & Weigle W. O. Lymphokines and aging: interleukin-2 production and activity in aged animals. J Immunol 127, 2102–2106 (1981). 6457862

[b45] Jagger A., Shimojima Y., Goronzy J. J. & Weyand C. M. Regulatory T cells and the immune aging process: a mini-review. Gerontology 60, 130–137, doi: 10.1159/000355303 (2014). 24296590 PMC4878402

[b46] Chougnet C. A. . A major role for Bim in regulatory T cell homeostasis. J Immunol 186, 156–163, doi: 10.4049/jimmunol.1001505 (2011). 21098226 PMC3066029

[b47] Bouillet P. . Proapoptotic Bcl-2 relative Bim required for certain apoptotic responses, leukocyte homeostasis, and to preclude autoimmunity. Science 286, 1735–1738 (1999). 10576740 10.1126/science.286.5445.1735

[b48] Herold M. J. . Foxo-mediated Bim transcription is dispensable for the apoptosis of hematopoietic cells that is mediated by this BH3-only protein. EMBO Rep 14, 992–998, doi: 10.1038/embor.2013.152 (2013). 24060902 PMC3818073

[b49] Kau A. L., Ahern P. P., Griffin N. W., Goodman A. L. & Gordon J. I. Human nutrition, the gut microbiome and the immune system. Nature 474, 327–336, doi: 10.1038/nature10213 (2011). 21677749 PMC3298082

[b50] Lozupone C., Lladser M. E., Knights D., Stombaugh J. & Knight R. UniFrac: an effective distance metric for microbial community comparison. ISME J 5, 169–172, doi: 10.1038/ismej.2010.133 (2011). 20827291 PMC3105689

[b51] Claesson M. J. . Composition, variability, and temporal stability of the intestinal microbiota of the elderly. Proc Natl Acad Sci USA 108 Suppl 1, 4586–4591, doi: 10.1073/pnas.1000097107 (2011). 20571116 PMC3063589

[b52] Mariat D. . The Firmicutes/Bacteroidetes ratio of the human microbiota changes with age. BMC Microbiol 9, 123, doi: 10.1186/1471-2180-9-123 (2009). 19508720 PMC2702274

[b53] Xiao S. . A gut microbiota-targeted dietary intervention for amelioration of chronic inflammation underlying metabolic syndrome. FEMS Microbiol Ecol 87, 357–367, doi: 10.1111/1574-6941.12228 (2014). 24117923 PMC4255291

[b54] Zweigner J., Schumann R. R. & Weber J. R. The role of lipopolysaccharide-binding protein in modulating the innate immune response. Microbes Infect 8, 946–952, doi: 10.1016/j.micinf.2005.10.006 (2006). 16483818

[b55] Huber V. C. . Distinct contributions of vaccine-induced immunoglobulin G1 (IgG1) and IgG2a antibodies to protective immunity against influenza. Clin Vaccine Immunol 13, 981–990, doi: 10.1128/CVI.00156-06 (2006). 16960108 PMC1563571

[b56] Harper J. M., Galecki A. T., Burke D. T. & Miller R. A. Body weight, hormones and T cell subsets as predictors of life span in genetically heterogeneous mice. Mechanisms of Ageing and Development 125, 381–390, doi: 10.1016/j.mad.2004.03.003 (2004). 15130756

[b57] Sharma S., Dominguez A. L. & Lustgarten J. High accumulation of T regulatory cells prevents the activation of immune responses in aged animals. J Immunol 177, 8348–8355 (2006). 17142731 10.4049/jimmunol.177.12.8348

[b58] Fink C. . Intestinal FoxO signaling is required to survive oral infection in Drosophila. Mucosal Immunol, doi: 10.1038/mi.2015.112 (2015). 26627459

[b59] Hesp K., Smant G. & Kammenga J. E. Caenorhabditis elegans DAF-16/FOXO transcription factor and its mammalian homologs associate with age-related disease. Exp Gerontol 72, 1–7, doi: 10.1016/j.exger.2015.09.006 (2015). 26363351

[b60] Lin L., Hron J. D. & Peng S. L. Regulation of NF-kappaB, Th activation, and autoinflammation by the forkhead transcription factor Foxo3a. Immunity 21, 203–213, doi: 10.1016/j.immuni.2004.06.016 (2004). 15308101

[b61] Morris B. J., Willcox D. C., Donlon T. A. & Willcox B. J. FOXO3: A Major Gene for Human Longevity–A Mini-Review. Gerontology 61, 515–525, doi: 10.1159/000375235 (2015). 25832544 PMC5403515

[b62] Claesson M. J. . Gut microbiota composition correlates with diet and health in the elderly. Nature 488, 178–184, doi: 10.1038/nature11319 (2012). 22797518

[b63] Perez Martinez G., Bauerl C. & Collado M. C. Understanding gut microbiota in elderly’s health will enable intervention through probiotics. Benef Microbes 5, 235–246, doi: 10.3920/BM2013.0079 (2014). 24889891

[b64] Thomas F., Hehemann J. H., Rebuffet E., Czjzek M. & Michel G. Environmental and gut bacteroidetes: the food connection. Front Microbiol 2, 93, doi: 10.3389/fmicb.2011.00093 (2011). 21747801 PMC3129010

[b65] Valdez Y., Brown E. M. & Finlay B. B. Influence of the microbiota on vaccine effectiveness. Trends Immunol 35, 526–537, doi: 10.1016/j.it.2014.07.003 (2014). 25113637

[b66] Biagi E. . Through ageing, and beyond: gut microbiota and inflammatory status in seniors and centenarians. PLoS One 5, e10667, doi: 10.1371/journal.pone.0010667 (2010). 20498852 PMC2871786

[b67] Drago L., Toscano M., Rodighiero V., De Vecchi E. & Mogna G. Cultivable and pyrosequenced fecal microflora in centenarians and young subjects. J Clin Gastroenterol 46 Suppl, S81–84, doi: 10.1097/MCG.0b013e3182693982 (2012). 22955365

[b68] Tsai Y. T., Cheng P. C. & Pan T. M. The immunomodulatory effects of lactic acid bacteria for improving immune functions and benefits. Appl Microbiol Biotechnol 96, 853–862, doi: 10.1007/s00253-012-4407-3 (2012). 23001058

[b69] Hori T., Kiyoshima J., Shida K. & Yasui H. Augmentation of cellular immunity and reduction of influenza virus titer in aged mice fed Lactobacillus casei strain Shirota. Clin Diagn Lab Immunol 9, 105–108 (2002). 11777838 10.1128/CDLI.9.1.105-108.2002 PMC119906

[b70] Bayry J. Regulatory T cells as adjuvant target for enhancing the viral disease vaccine efficacy. Virusdisease 25, 18–25, doi: 10.1007/s13337-013-0187-3 (2014). 24426307 PMC3889236

[b71] Wang S. M., Tsai M. H., Lei H. Y., Wang J. R. & Liu C. C. The regulatory T cells in anti-influenza antibody response post influenza vaccination. Hum Vaccin Immunother 8, 1243–1249, doi: 10.4161/hv.21117 (2012). 22894960 PMC3579905

[b72] Shin N. R., Whon T. W. & Bae J. W. Proteobacteria: microbial signature of dysbiosis in gut microbiota. Trends Biotechnol 33, 496–503, doi: 10.1016/j.tibtech.2015.06.011 (2015). 26210164

[b73] Oh J. Z. . TLR5-mediated sensing of gut microbiota is necessary for antibody responses to seasonal influenza vaccination. Immunity 41, 478–492, doi: 10.1016/j.immuni.2014.08.009 (2014). 25220212 PMC4169736

[b74] Bordone L. & Guarente L. Calorie restriction, SIRT1 and metabolism: understanding longevity. Nat Rev Mol Cell Biol 6, 298–305, doi: 10.1038/nrm1616 (2005). 15768047

[b75] Kitada M. & Koya D. SIRT1 in Type 2 Diabetes: Mechanisms and Therapeutic Potential. Diabetes Metab J 37, 315–325, doi: 10.4093/dmj.2013.37.5.315 (2013). 24199159 PMC3816131

[b76] Stephenson I., Wood J. M., Nicholson K. G., Charlett A. & Zambon M. C. Detection of anti-H5 responses in human sera by HI using horse erythrocytes following MF59-adjuvanted influenza A/Duck/Singapore/97 vaccine. Virus Res 103, 91–95, doi: 10.1016/j.virusres.2004.02.019 (2004). 15163495

[b77] Chun J., Kim K. Y., Lee J. H. & Choi Y. The analysis of oral microbial communities of wild-type and toll-like receptor 2-deficient mice using a 454 GS FLX Titanium pyrosequencer. BMC Microbiol 10, 101, doi: 10.1186/1471-2180-10-101 (2010). 20370919 PMC2873484

[b78] Finn R. D., Clements J. & Eddy S. R. HMMER web server: interactive sequence similarity searching. Nucleic Acids Res 39, W29–37, doi: 10.1093/nar/gkr367 (2011). 21593126 PMC3125773

[b79] Kim O. S. . Introducing EzTaxon-e: a prokaryotic 16S rRNA gene sequence database with phylotypes that represent uncultured species. Int J Syst Evol Microbiol 62, 716–721, doi: 10.1099/ijs.0.038075-0 (2012). 22140171

[b80] Schloss P. D. . Introducing mothur: open-source, platform-independent, community-supported software for describing and comparing microbial communities. Appl Environ Microbiol 75, 7537–7541, doi: 10.1128/AEM.01541-09 (2009). 19801464 PMC2786419

