# Corrigendum: Modulation of gut microbiota and delayed immunosenescence as a result of syringaresinol consumption in middle-aged mice

**DOI:** 10.1038/srep41667

**Published:** 2017-01-31

**Authors:** Si-Young Cho, Juewon Kim, Ji Hae Lee, Ji Hyun Sim, Dong-Hyun Cho, Il-Hong Bae, Hyunbok Lee, Min A. Seol, Hyun Mu Shin, Tae-Joo Kim, Dae-Yong Kim, Su-Hyung Lee, Song Seok Shin, Sin-Hyeog Im, Hang-Rae Kim

Scientific Reports 6: Article number: 39026; 10.1038/srep39026 published online: 12 15 2016; updated: 01 31 2017.

The original version of this Article contained an error in the spelling of Sin-Hyeog Im, which was incorrectly given as Sin-Hyeog Lm. This has now been corrected in the PDF and HTML versions of this Article.

