# Supplementary material for: Modulation of gut microbiota and delayed immunosenescence as a result of syringaresinol consumption in middle-aged mice
Source: Sci Rep. 2016 Dec 15;6:39026. doi: 10.1038/srep39026 (PMC5157019; doi:10.1038/srep39026)
Supplement: Supplementary Information [file srep39026-s1.pdf]

Modulation of gut microbiota and delayed immunosenescence as a result of syringaresinol consumption in middle-aged mice

Si-Young Cho[1], Juewon Kim[1], Ji Hae Lee1, Ji Hyun Sim[2], Dong-Hyun Cho[1], Il-Hong Bae[1], Hyunbok Lee[1], Min A Seol[2,3], Hyun Mu Shin[2,3,4], Tae-Joo Kim[2,3], Dae-Yong Kim[5], Su-Hyung Lee[5], Song Seok Shin1, Sin-Hyeog lm[6,7*] and Hang-Rae Kim[2,3,4*]

[1] R&D Unit, AmorePacific Corporation, Gyeonggi-do 17074, Republic of Korea
[2]Department of Anatomy and Cell Biology, [3]Department of Biomedical Sciences, and [4]BK21Plus Biomedical Science Project, Seoul National University College of Medicine, Seoul 03080, Republic of Korea
[5]College of Veterinary Medicine, Seoul National University, Seoul 08826, Republic of Korea
[6]Academy of Immunology and Microbiology (AIM), Institute for Basic Science (IBS), Pohang, 790-784, Republic of Korea.
[7]Division of Integrative Biosciences and Biotechnology (IBB), Pohang University of Science and Technology, Pohang, 790-784, Republic of Korea.

*Correspondence:
Hang-Rae Kim, D.V.M., Ph.D.
Department of Anatomy and Cell Biology, Department of Biomedical Biosciences, Seoul National University College of Medicine, 103 Daehak-ro, Jongno-gu, Seoul 03080, Republic of Korea. Phone: (82) 2-740-8214, Fax: (82) 2-745-9528, E-mail: hangrae2@snu.ac.kr

or

Sin-Hyeog lm, Ph.D.
Address: Academy of Immunology and Microbiology (AIM), Institute for Basic Science (IBS), Academy of Immunology and Microbiology (AIM), Institute for Basic Science (IBS), Pohang, Republic of Korea; Division of Integrative Biosciences and Biotechnology (IBB), Pohang University of Science and Technology, Pohang, Republic of Korea 790-784; Tel: 82-54-279-2356; FAX: 82-54-279-8768; Email: iimsh@postech.ac.kr

Figure S1

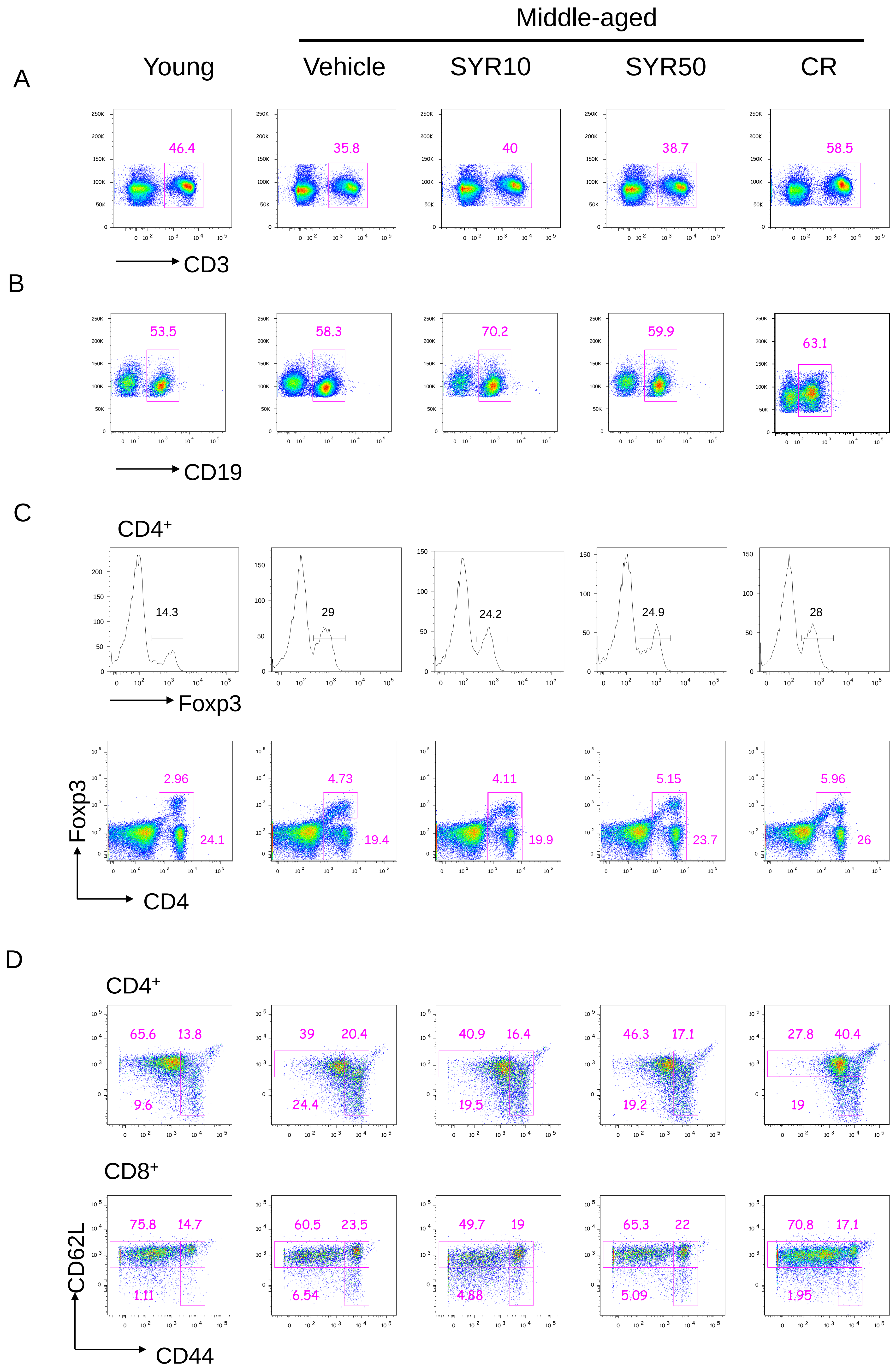

Figure S1. Representative dot plots of lymphocyte subsets from spleen.
Middle-aged mice (each group n = 6) were treated with vehicle (control) 10 mg/kg SYR, 50 mg/kg SYR or CR for 10 weeks and their splenocytes analyzed by flow cytometry. Young mice were used as age-mismatched controls. Representative flow cytometric data for frequencies of (A) $CD3^+$ T cells, (B) $CD19^+$ B cells, (C) $foxp3^+$ Treg of $CD4^+$ T cells, and (D) T-cell subsets, such as naïve T cells ($CD62L^+CD44^-$), central memory T cells ($CD62L^+CD44^+$), and effector memory T cells ($CD62L^-CD44^+$) are shown.

Figure S2

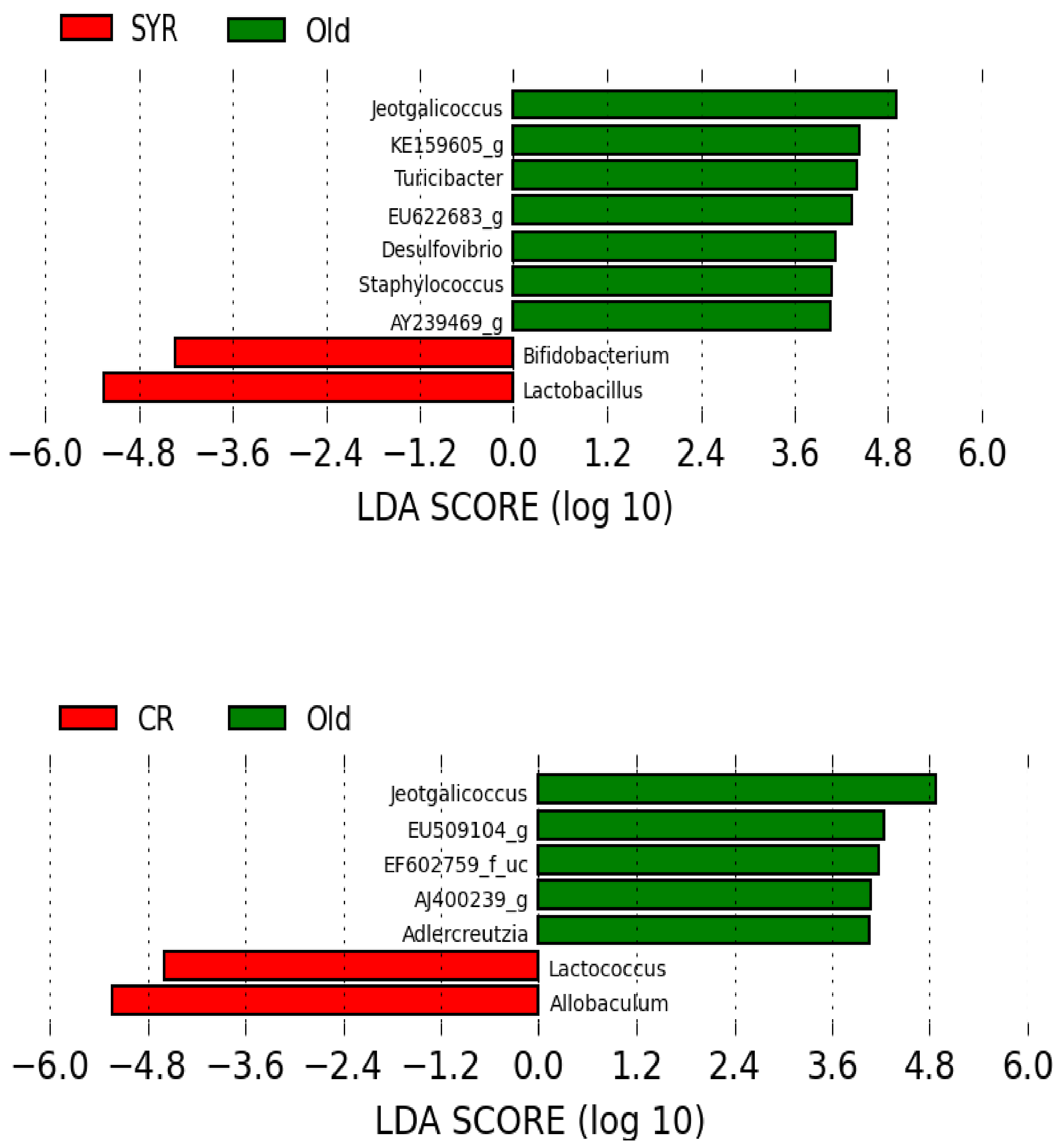

Figure S2. Key phylotypes of the microbiota of cecal contents of middle-aged mice (A) Taxonomic differences between vehicle control and 50 mg/kg SYR-treated middle-aged mice. (B) Taxonomic differences between vehicle control and CR-treated middle-aged mice. Significant differences in LDA scores ($p < 0.05$) were evident among genera (Kruskal–Wallis test) and between subclasses (Wilcoxon’s test). The threshold logarithmic LDA score was 4.0.

Table S1. Effects of SYR and CR treatment on body weight and glucose levels.

	Vehicle	SYR 10	SYR 50	CR
Initial Body weight (g)	35.5 ± 0.94	35.39 ± 0.91	35.53 ± 0.77	35.5 ± 0.67
Body weight gain (g)	-0.34 ± 0.92	-0.24 ± 1.02	-0.16 ± 0.85	-10.33 ± 0.55***
Food intake (g/day)	3.57 ± 0.03	3.61 ± 0.03	3.57 ± 0.02	2.47 ± 0***
Glucose (mg/dl)	129. 3 ± 5.69	124 ± 5.29	110.8 ± 4.57	100 ± 2.91**

Middle-aged mice (44 weeks old) were subjected to vehicle (control), 10 mg/kg SYR (SYR 10), 50 mg/kg SYR (SYR 50) or 30% CR treatment for 10 weeks (n = 6, group). All results are expressed as means ± SEM. Statistical analyses were performed using one-way ANOVA followed by Dunnett's post hoc test. ** $P < 0.01$, ***$P < 0.001$ versus the untreated middle-aged control group. Experiments were repeated three times. Data are the averages of three independent experiments.

Table S2. Frequencies of splenic leukocyte subpopulations.

**Frequency (%)** *P* **value**	**Young mice**	**Middle-aged mice**			
		**Vehicle**	**SYR10**	**SYR50**	**CR**
$CD3^+$ T cells	43 ± 1.3<br>***	33.8 ± 2.1	37.7 ± 1.8<br>ns	41.5 ± 1.2<br>***	50.0 ± 2.4<br>***
$CD3^+CD4^+$	24.2 ± 0.7<br>***	16.8 ± 0.7	19.9 ± 0.7<br>*	21.2 ± 0.6<br>***	17.1 ± 0.8<br>ns
$CD3^+CD8^+$	16.5 ± 1.0<br>*	12.9 ± 0.6	14.9 ± 1.0<br>ns	16.3 ± 0.5<br>*	29.0 ± 1.0<br>***
$CD4^+/CD3^+$	55.1 ± 1.0<br>ns	51.2 ± 0.5	52.0 ± 1.0<br>ns	51.3 ± 0.5<br>ns	34.2 ± 1.9<br>***
$CD8^+/CD3^+$	38.1 ± 1.1<br>ns	39.3 ± 0.6	40.4 ± 1.0<br>ns	40.8 ± 0.4<br>ns	57.6 ± 1.3<br>***
Naïve/$CD4^+$	72.3 ± 0.9<br>***	47.23 ± 1.5	50.3 ± 0.7<br>ns	53.6 ± 1.0<br>**	64.8 ± 1.4<br>***
Naïve/$CD8^+$	72.0 ± 0.9<br>***	55.4 ± 1.5	55.4 ± 2.3<br>ns	63.1 ± 0.8<br>*	72.2 ± 2.1<br>***
$Foxp3^+CD4^+$ (Tregs)	2.36 ± 0.1<br>***	4.29 ± 0.4	3.7 ± 0.2<br>ns	3.9 ± 0.1<br>ns	3.6 ± 0.3<br>ns
Treg/$CD4^+$	13.9 ± 0.5<br>***	34.7 ± 3.2	24.9 ± 0.7<br>**	24.0 ± 0.2<br>**	28.7 ± 2.33<br>ns
$CD19^+$ B cells	33.7 ± 1.3<br>***	47.8 ± 1.5	37.9 ± 1.6<br>***	36.6 ± 1.0<br>***	30.9 ± 1.6<br>***
Transitional B/$CD19^+$	15.5 ± 1.1<br>***	5.7 ± 0.2	6.05 ± 0.3<br>ns	6.0 ± 0.7<br>ns	3.5 ± 0.3<br>ns
FOB/$CD19^+$	47.1 ± 3.2<br>***	62.8 ± 0.8	65.4 ± 1.0<br>ns	63.4 ± 1.1<br>ns	57.6 ± 2.3<br>ns
MZB/$CD19^+$	7.59 ± 1.2<br>**	13.7 ± 1.0	13.7 ± 1.3<br>ns	14.4 ± 0.9<br>ns	22.1 ± 1.7<br>***
Dendritic cells ($CD11c^+$)	2.03 ± 0.1<br>ns	2.02 ± 0.15	2.07± 0.10<br>ns	2.30± 0.3<br>ns	1.17 ± 0.10<br>**
Monocytes ($CD11b^+Ly6C^+$)	0.27 ± 0.05<br>ns	0.28 ± 0.04	0.30 ±0.03<br>ns	0.28± 0.02<br>ns	0.37 ± 0.06<br>ns
Granulocytes $CD11b^+Ly6G^+$	1.06 ± 0.3<br>ns	0.47 ± 0.10	0.57 ± 0.1<br>ns	0.37± 0.06<br>ns	0.41 ± 0.07<br>ns

Middle-aged mice were subjected to vehicle (control), 10 mg/kg SYR (SYR 10), 50 mg/kg SYR (SYR 50) or 30% CR treatment for 10 weeks (n=6, group). Young mice were used as age-mismatched controls. All data are expressed as means ± SEM. Statistical analysis were performed using one-way ANOVA followed by Dunnett's post hoc test. * $P < 0.05$, ** $P < 0.01$, *** $P < 0.001$ versus the untreated middle-aged control group; ns, non-significant. Transitional B; $CD21^+CD23^+$, follicular B cells (FOB); $CD21^+CD23^{++}$, marginal zone B cells

(MZB); $CD21^{++}CD23^{+}$.

Table S3. Numbers of splenic leukocyte subpopulations.

**Number**<br>***P* value**	**Young mice**	**Middle-aged mice**			
		**Vehicle**	**SYR10**	**SYR50**	**CR**
Splenocytes (×10^7)	20.3 ± 2.6<br>***	38.6 ± 2.4	35.7 ± 1.1<br>ns	30.2 ± 2.3<br>*	6.56 ± 0.7<br>***
$CD3^+$ (×10^6)	16.2 ± 2.4<br>ns	18.7 ± 2.4	23.4 ± 1.0<br>ns	22.1 ± 1.4<br>ns	8.1 ± 0.8<br>***
$CD3^+CD4^+$ (×10^6)	9.01 ± 1.4<br>ns	9.7 ± 1.1	12.2 ± 0.8<br>ns	11.7 ± 0.9<br>ns	2.6 ± 0.4<br>***
$CD3^+CD8^+$ (×10^6)	6.09 ± 0.8<br>ns	7.48 ± 0.9	9.1 ± 0.5<br>ns	9.05 ± 0.5<br>ns	4.3 ± 0.4<br>**
Treg (×10^6)	0.475 ± 0.06<br>***	1.56 ± 0.14	1.40 ± 0.12<br>ns	1.19 ± 0.10<br>*	0.23 ± 0.02<br>***
$CD19^+$ (×10^6)	14.2 ± 2.4<br>***	30.5 ± 2.6	26.6 ± 2.9<br>ns	22.9 ± 2.8<br>ns	4.8 ± 0.7<br>***
Dendritic cells (×10^6)	0.61 ± 0.10<br>ns	1.06 ± 0.12	1.09 ± 0.12<br>ns	1.08 ± 0.26<br>ns	0.12 ± 0.01<br>***
$CD11b^+Ly6C^+$ (×10^6)	0.08 ± 0.02<br>ns	0.15 ± 0.03	0.14 ± 0.02<br>ns	0.13 ± 0.02<br>ns	0.04 ± 0.01<br>**
$CD11b^+Ly6G^+$ (×10^6)	0.36 ± 0.1<br>ns	0.26 ± 0.07	0.28 ± 0.05<br>ns	0.16 ± 0.02<br>ns	0.05 ± 0.01<br>ns

Middle-aged mice were subjected to vehicle (control), 10 mg/kg SYR (SYR 10), 50 mg/kg SYR (SYR 50) or 30% CR treatment for 10 weeks (n=6, group). Young mice were used as age-mismatched controls. All data are expressed as means ± SEM. Statistical analysis were performed using one-way ANOVA followed by Dunnett's post hoc test. * $P < 0.05$, ** $P < 0.01$, *** $P < 0.001$ versus the untreated middle-aged control group; ns, non-significant.

Table S4. Summary of analysis of sequences obtained from cecal contents.

	Treat	Number of subjects	Analyzed reads	Normalized reads	Observed OTUs	Estimated OTUs (Chao1)	Shannon diversity index	Good's coverage
Young mice		Y1	5245	3600	576	1117.8	4.95	0.92
		Y2	5162	3600	424	898	4.33	0.93
		Y3	3760	3600	578	1282.2	5.01	0.92
		Y4	5069	3600	443	712.9	4.30	0.94
		Y5	4803	3600	559	1153.8	4.58	0.91
		Y6	5579	3600	521	1171	3.99	0.92
						1056±85.72	4.53±0.162	
Middle-aged mice	Vehicle	O1	6273	3600	211	356.5	2.63	0.97
		O2	5904	3600	275	408.2	3.72	0.97
		O3	5281	3600	454	701.6	4.58	0.94
		O4	3847	3600	359	570.5	4.46	0.96
		O5	4548	3600	397	581.7	4.45	0.96
		O6	5812	3600	326	574.1	3.84	0.96
						532.1±51.84	3.95±0.301	
	SYR 10	SYRL1	3413	2147	424	757.8	5.08	0.90
		SYRL2	3427	2147	428	765.6	4.73	0.90
		SYRL3	3925	3600	387	695	4.32	0.95
		SYRL4	2147	2147	373	532.4	4.83	0.93
		SYRL5	3967	3600	424	685.9	4.48	0.95
		SYRL6	2286	2147	329	581.9	4.76	0.93
						669.8±38.48	4.70±0.109	
	SYR50	SYRH1	4073	3600	141	233.8	2.79	0.98
		SYRH2	3817	3600	195	304.4	2.82	0.98
		SYRH3	3148	2147	138	238.8	3.07	0.97
		SYRH4	3605	3600	197	296.6	3.03	0.98
		SYRH5	2535	2147	186	380.1	3.01	0.96
		SYRH6	3628	3600	259	443.0	3.81	0.97
						316.1±33.40	3.09±0.152	
	CR	CR1	5132	3600	272	496.1	3.75	0.96
		CR2	4746	3600	200	308	3.15	0.98
		CR3	4537	3600	255	389.6	3.17	0.97
		CR4	4415	3600	411	717.2	4.26	0.94
		CR5	4219	3600	249	431.8	3.31	0.97
		CR6	2850	2147	204	338.1	3.48	0.96
						446.8±60.59	3.52±0.174	

Middle-aged mice were subjected to vehicle (control), 10 mg/kg SYR (SYR 10), 50 mg/kg SYR (SYR 50) or 30%

CR treatment for 10 weeks (n=6, group). Young mice were used as age-mismatched controls. Mean ± SEM.

Table S5. Relative abundance (% of total 16S rDNA) of the most representative genus in cecal contents.

Taxon	Young mice	Middle-aged mice			
		Vehicle	SYR10	SYR50	CR
Firmicutes;;Bacilli;;Lactobacillales;;Lactobacillaceae;;Lactobacillus	23.8 ± 3.46	2.13 ± 0.46	1.6 ± 0.82	38.3 ± 7.8	1.80 ± 0.62
	ns		ns	0.022	ns
Firmicutes;;Erysipelotrichi;;Erysipelotrichales;;Allobaculum_f;;Allobaculum	0	2.01 ± 1.55	0.03 ± 0.03	16.97 ± 10.8	35. 7± 5.64
	ns		ns	ns	0.0022
Firmicutes;;Erysipelotrichi;;Erysipelotrichales;;Allobaculum_f;;EF603943_g	0	10.8 ± 5.43	10.80 ± 3.99	19.35 ± 8.16	2.02 ± 0.48
	0.0088		ns	ns	ns
Firmicutes;;Bacilli;;Bacillales;;Staphylococcaceae;;Jeotgalicoccus	0.06 ± 0.05	19.75 ± 6.99	1.43 ± 0.92	1.02 ± 0.82	5.81 ± 2.24
	0.0007		0.0057	0.0431	ns
Firmicutes;;Clostridia;;Clostridiales;;Lachnospiraceae;;Hungatella	7.19 ± 0.99	3.68 ± 1.57	7.58 ± 1.43	0.97 ± 0.38	2.0 ± 0.36
	ns		ns	ns	ns
Firmicutes;;Clostridia;;Clostridiales;;Lachnospiraceae;;AB626958_g	3.68 ± 0.65	5.22 ± 1.85	4.44 ± 0.69	0.79 ± 0.25	1.42 ± 0.54
	ns		ns	ns	ns
Firmicutes;;Clostridia;;Clostridiales;;Lachnospiraceae;;Lachnospiraceae_uc	9.65 ± 2.42	1.43 ± 0.42	0.39 ± 0.35	0.38 ± 0.14	1.20 ± 0.31
	0.0192		ns	ns	ns
Firmicutes;;Erysipelotrichi;;Erysipelotrichales;;Erysipelotrichales_uc;;Erysipelotrichales_uc_g	0	2.84 ± 1.99	4.96 ± 1.80	4.40 ± 1.91	8.67 ± 3.72
	ns		ns	ns	ns
Proteobacteria;;Deltaproteobacteria;;Desulfovibrionales;;Desulfovibrionaceae;;Desulfovibrio	0	2.85 ± 0.67	2.30 ± 0.42	0.19 ± 0.07	8.21 ± 2.39
	0.0022		ns	0.0022	ns
Firmicutes;;Clostridia;;Clostridiales;;Lachnospiraceae;;KE159605_g	3.56 ± 1.54	5.00 ± 4.44	2.54 ± 1.03	0.16 ± 0.05	0.58 ± 0.12

	ns		ns	0.0281	ns
Firmicutes;;Erysipelotrichi;;Turicibacter_o;;Turicibacter_f;;Turicibacter	0.05 ± 0.03	4.52 ± 2.23	4.84 ± 0.77	0.02 ± 0.02	4.46 ± 1.79
	ns		ns	0.0363	ns
Bacteroidetes;;Bacteroidia;;Bacteroidales;;EF602759_f;;EU622683_g	0	3.86 ± 2.36	3.71 ± 3.12	0	2.31 ± 1.02
	0.0248		ns	0.0248	ns
Verrucomicrobia;;Verrucomicrobiae;;Verrucomicrobiales;;Akkermansia_f;;Akkermansia	0.11 ± 0.09	2.41 ± 1.34	0.14 ± 0.08	0.44 ± 0.33	3.84 ± 3.10
	ns		ns	ns	ns
Firmicutes;;Erysipelotrichi;;Erysipelotrichales;;Allobaculum_f;;Allobaculum_f_uc	0	3.34 ± 3.05	0	1.66 ± 0.51	4.01 ± 0.57
	ns		ns	ns	ns
Bacteroidetes;;Bacteroidia;;Bacteroidales;;Bacteroidaceae;;Bacteroides	8.37 ± 2.98	0.02 ± 0.2	0.62 ± 0.44	0	0
	0.0022		ns	ns	ns
Actinobacteria;;Actinobacteria_c;;Bifidobacteriales;;Bifidobacteriaceae;;Bifidobacterium	0.01 ± 0	1.19 ± 0.53	0	5.60 ± 1.1	1.94 ± 0.95
	ns		ns	0.065	ns

Middle-aged mice were subjected to vehicle (control), 10 mg/kg SYR (SYR 10), 50 mg/kg SYR (SYR 50) or 30% CR treatment for 10 weeks (n=6, group). Young mice were used as age-mismatched controls. Results are expressed as means ± SEM of the relative abundance (% of total 16S rDNA) of the genera detected in cecal samples by 16S rDNA pyrosequencing. Statistical analyses were performed using the Kruskal–Wallis test followed by Mann–Whitney U-test. *P* values were obtained by comparison of young, SYR 10, SYR 50 and CR groups with the untreated middle-aged control group; ns, non-significant.

Table S6. Pharmacokinetic properties of SYR.

	I.V. (1 mg/kg)	Oral (2 mg/kg)	Oral (20 mg/kg)
tmax (h)	-	0.17 ± 0.0	0.63 ± 0.9
Cmax (ng/mL)	-	115.0 ± 27.3	776.2 ± 205.3
$AUC_{(0-t)}$ (ng·h/mL)	165.1 ± 32.8	197.2 ± 130.6	2,436.5 ± 561.2
$AUC_{0-\infty}$ (ng·h/mL)	166.0 ± 33.5	-	-
$t_{1/2}$ (h)	0.16 ± 0.02	-	-
